# The relative importance of reproduction and survival for the conservation of two dolphin populations

**DOI:** 10.1002/ece3.2130

**Published:** 2016-04-20

**Authors:** Oliver Manlik, Jane A. McDonald, Janet Mann, Holly C. Raudino, Lars Bejder, Michael Krützen, Richard C. Connor, Michael R. Heithaus, Robert C. Lacy, William B. Sherwin

**Affiliations:** ^1^ Evolution and Ecology Research Centre School of Biological, Earth and Environmental Sciences University of New South Wales Sydney New South Wales 2052 Australia; ^2^ Department of Biology and Psychology Georgetown University 37th and O St. NW Washington DC 20057; ^3^ Cetacean Research Unit School of Veterinary and Life Sciences Murdoch University South Street Murdoch Western Australia 6150 Australia; ^4^ Marine Science Program Department of Parks and Wildlife 17 Dick Perry Avenue. Perth Western Australia 6151 Australia; ^5^ Anthropological Institute and Museum University of Zurich Winterthurerstrasse 190 8057 Zurich Switzerland; ^6^ Biology Department UMASS‐Dartmouth Dartmouth Massachusetts 02747; ^7^ Department of Biological Science School of Environment Arts and Society Florida International University North Miami Florida 33181; ^8^ Chicago Zoological Society Brookfield Illinois 60513; ^9^ Present address: School of Biological Sciences University of Queensland St Lucia Queensland 4067 Australia

**Keywords:** Bottlenose dolphin, cetacean, population dynamics, population viability, PVA, sensitivity analysis, Tursiops, wildlife management

## Abstract

It has been proposed that in slow‐growing vertebrate populations survival generally has a greater influence on population growth than reproduction. Despite many studies cautioning against such generalizations for conservation, wildlife management for slow‐growing populations still often focuses on perturbing survival without careful evaluation as to whether those changes are likely or feasible. Here, we evaluate the relative importance of reproduction and survival for the conservation of two bottlenose dolphin (*Tursiops* cf *aduncus*) populations: a large, apparently stable population and a smaller one that is forecast to decline. We also assessed the feasibility and effectiveness of wildlife management objectives aimed at boosting either reproduction or survival. Consistent with other analytically based elasticity studies, survival had the greatest effect on population trajectories when altering vital rates by equal proportions. However, the findings of our alternative analytical approaches are in stark contrast to commonly used proportional sensitivity analyses and suggest that reproduction is considerably more important. We show that

1. in the stable population reproductive output is higher, and adult survival is lower;
2. the difference in viability between the two populations is due to the difference in reproduction;
3. reproductive rates are variable, whereas survival rates are relatively constant over time;
4. perturbations on the basis of observed, temporal variation indicate that population dynamics are much more influenced by reproduction than by adult survival;
5. for the apparently declining population, raising reproductive rates would be an effective and feasible tool to reverse the forecast population decline; increasing survival would be ineffective.

Our findings highlight the importance of reproduction – even in slow‐growing populations – and the need to assess the effect of natural variation in vital rates on population viability. We echo others in cautioning against generalizations based on life‐history traits and recommend that population modeling for conservation should also take into account the magnitude of vital rate changes that could be attained under alternative management scenarios.

## Introduction

Given limited resources and time for conservation interventions, it is crucial to focus wildlife management recommendations on vital rates that have the greatest impact on population viability. Unfortunately, for many species and populations of conservation concern, demographic data are scarce, compelling scientists to make generalizations based on shared life‐history traits (Heppell et al. 2000; van de Kerk et al. 2013).

Several studies have shown that in slow‐growing populations, characterized by late maturation, small litter size, and long life spans, population growth is influenced more by adult survival than by reproduction (Heppell et al. 2000; Crone 2001; Oli and Dobson 2003). This finding seems to hold true for long‐lived bird species with low clutch size (Saether and Bakke 2000) and mammals with slow population growth, including cetaceans (Brault and Caswell 1993; Young and Edward 2011). Consequently, adult survival has often been regarded as more important than reproduction to the viability of slow‐growing populations: Lebreton and Clobert (1991) argued that any observed changes in population growth rates of species with slow growth are likely due to changes in adult survival. Crone (2001), who conducted a meta‐sensitivity analysis on over one hundred taxa, suggested that for slow‐growing populations, survival is generally a better fitness surrogate than reproduction. Although this has been questioned (e.g., Mills and Lindberg 2002), such generalizations for conservation on the basis of shared life histories are still common. In a recent meta‐analysis of 27 carnivore taxa, van de Kerk et al. (2013) proposed a “rule of thumb,” suggesting that “for a slow reproducer […] conservation strategies targeted on adult survival are expected to be more effective” because “growth rate of slow species generally has a high elasticity for adult survival” (p. 7).

However, the reliability of conservation actions based on infinitesimal analytical sensitivity and elasticity analyses has been challenged by a number of studies (e.g., Mills et al. 1999; Wisdom et al. 2000; Morris and Doak 2002). This is because the importance of vital rates for conservation depends both on its infinitesimal effect (captured by analytical sensitivity and elasticity) and on how much vital rates actually vary, as well as how much they can be manipulated by management actions. A relatively large reduction or smaller, but simultaneous reductions of multiple vital rates with low elasticities (e.g., reproduction or calf survival in our study) can lead to similarly large decreases in growth rates compared to a small change in a vital rate with high elasticity (i.e., adult survival) (Biek et al. 2002). Therefore, the sensitivity of growth rates to proportional changes in reproduction and survival gives few clues to wildlife managers regarding the effectiveness and feasibility of management actions. Two crucial questions for wildlife managers are as follows: (1) Is a given management action aimed at increasing reproduction or survival feasible and attainable? In other words, is it possible to increase reproduction or survival by X% with a given management option? (2) If the proposed action is feasible, is population viability improved more effectively by increasing reproduction or survival? Proportional sensitivity analyses alone do not provide answers to these questions.

Consequently, in order to evaluate feasibility and effectiveness of wildlife management objectives, alternative analytical approaches are required. Such an approach could include the analysis of natural variability of vital rates and identifying vital rates that are depressed below taxon‐typical rates. Natural variability of vital rates can offer clues with respect to feasibility of management scenarios because a scenario that aims to increase a given vital rate beyond its natural fluctuation may be unattainable. Numerous studies have previously shown that reproductive rates of slow‐growing populations tend to vary much more than survival rates (Pfister 1998; Gaillard et al. 2000; Raithel et al. 2007; Mitchell et al. 2009). Furthermore, several studies showed that the larger variability in reproductive rates can also translate to having a greater impact on population viability (e.g., Saether and Bakke 2000; Raithel et al. 2007; Mitchell et al. 2009). This effect of observed vital rate fluctuations is often population‐specific (Mitchell et al. 2009; Johnson et al. 2010) and does not necessarily extrapolate to other populations or taxa.

Effectiveness of wildlife management actions also depends on which vital rates are depressed relative to observed rates, and their fluctuations, in stable populations. This is illustrated by a study on a marbled murrelet (*Brachyramphus marmoratus*) population, which is representative of a slow‐growing bird taxon. Prior to large‐scale population declines (1892–1922), reproductive rates were 8–9 times larger than contemporary rates, while adult survival rates remained at similar rates (Beissinger and Peery 2007). Therefore, such populations, which display constant and relatively high survival rates, are unlikely to be rescued by management actions aimed at increasing survival, regardless of the high sensitivity of population growth to proportional changes in survival. Furthermore, targeting survival instead of reproduction is not necessarily the most cost‐effective management option (Baxter et al. 2006).

The importance of vital rates is commonly quantified by sensitivity analyses, which have been widely used to rank alternative wildlife management strategies according to the relative influence of vital rates on population growth (reviewed in Mills and Lindberg 2002). The general principle of sensitivity analyses for population viability is to simulate the perturbation of different vital rates and to assess the effect of those changes on population dynamics – typically growth rate. There are two very different approaches to sensitivity analysis with respect to how each vital rate is changed: (1) each vital rate is changed by the same proportion (e.g., by 1%) – without reference to whether those changes are likely or feasible. We hereafter refer to this commonly used method as “fixed‐proportion analysis”; (2) perturbation of vital rates is based on observed variation over time – termed “observed‐variation analysis” herein. The fixed‐proportion and observed‐variation analysis are similar, but not identical to the “prospective analysis” and “retrospective analysis”, respectively, described by Caswell (2000). Examples of observed‐variation analysis include random sampling of values from the natural distribution (McCarthy et al. 1995), life‐stage analysis, based on random drawings of vital rates from specified probability distributions within the observed range of the vital rate (Wisdom et al. 2000), or alteration of the variable by ± one standard deviation.

The primary aim of our study was to assess the relative importance of reproduction and survival for conservation with an evaluation of feasibility and effectiveness of wildlife management objectives. We investigated two well‐studied free‐ranging bottlenose dolphin (*Tursiops* cf. *aduncus)* populations – representative of a taxon with slow population growth – in SB (Shark Bay) and Bunbury (Western Australia; see Appendix S1). In contrast to the relatively unimpacted population at SB, the Bunbury population inhabits waters adjacent to a regional city center and port with higher human activity. In order to investigate the importance of reproduction versus survival, we compared the demography and viability of the two populations and performed fixed‐proportion and observed‐variation analyses. In order to evaluate the effectiveness of wildlife management options aimed at either raising reproduction or survival, we tested whether various scenarios could potentially reverse a forecast population decline.

## Materials and Methods

### Demography and model parameterization

We conducted PVAs (population viability analyses) and sensitivity analyses with the software program vortex (version 9.99b, 19 May 2010; available at www.vortex10.org/Vortex10.aspx) (Lacy 1993: Lacy et al. 2005), which is best suited for taxa with low fecundity (Miller and Lacy 2005). The vortex program is an age‐based Monte Carlo simulation of stochastic and deterministic effects on the viability of populations (Lacy 1993). Detailed information on the structure of the vortex program can be found in Lacy (2000).

We estimated demographic parameters for two bottlenose dolphin populations in Western Australia. Estimates for input parameters for the SB model came from the Shark Bay Dolphin Research Project dataset (http://www.monkeymiadolphins.org/), including behavioral, ecological (1988–1999; Mann et al. 2000; Stanton and Mann 2012), and genetic data (1994–2003; Krützen et al. 2003, 2004a,b); this is one of the largest dolphin datasets in the world, with high survey intensity, and photo‐identification of over 1600 individuals.

We estimated input parameters for the SB model from individuals sighted within a 300 km^2^ area in the eastern gulf of SB (Fig. S1A) during four consecutive three‐year observation periods (1988–1990; 1991–1993; 1994–1996; and 1997–1999). The SB sampling effort included surveys of 12,463 groups and sightings of 1150 individuals. Initial population size (*N*_0_) was estimated as 2888 (SE ± 434) based on abundance and error estimates from repeated aerial surveys over 5 years by Preen et al. (1997). We set the carrying capacity to 4000 to allow population expansion (Table 1; Appendix S2).

**Table 1:** Main data from which input for vortex standard models were derived

		Shark Bay	Bunbury
Population structure			
Population subdivision		2 Subpopulations: East & West	NA
Number of individuals dispersing/three years		1.50^1^	NA
Initial population size		2888^2^ (East: 1444; West: 1444)	267
Carrying capacity		4000 (East: 2000; West: 2000)	370
Age class distribution (%)			
Calves		14.67 (*N* = 54)	16.87 (*N* = 41)
Juveniles		30.16 (*N* = 111)	24.69 (*N* = 60)
Adults		55.16 (*N* = 203)	58.44 (*N* = 142)
Sex ratio used for distribution of age classes		50:50 (male:female)	45:55 (male:female)
Reproductive system			
Female maturity at age category (age)		>4 (>12 years)	>4 (>12 years)
Male maturity at age category (age)		>5 (>15 years)	>5 (>15 years)
Maximum age category (age)		10 (30–33 years)	10 (30–33 years)
Sex ratio at birth		50:50 (male:female)	50:50 (male:female)
Three‐year reproductive rates (%)^3^		58.35 (SD_EV_ 9.38; CV_EV_ 0.161)	40.74 (SD_EV_ 13.54; CV_EV_ 0.332)
Males in breeding pool (%)		56.5	56.5
Three‐year survival rates (%)^3^			
Calves		73.48 (SD_EV_ 3.36; CV_EV_ 0.046)	71.67 (SD_EV_ 3.60; CV_EV_ 0.050)
Juve‐1^4^		95.71 (SD_EV_ 2.28; CV_EV_ 0.024)	
Juve‐2^4^	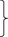 Juveniles^4^	98.94 (SD_EV_ 1.23; CV_EV_ 0.012) 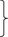 97.21	90.91 (SD_EV_ 2.79; CV_EV_ 0.031)
Subadults^4^		96.92 (SD_EV_ 2.66; CV_EV_ 0.027)	
Adults		90.28 (SD_EV_ 1.40; CV_EV_ 0.016)	95.95 (SD_EV_ 0.58; CV_EV_ 0.006)
Annual vital rates (%) Bunbury^5^			
Reproductive rate		NA	13.58 (SD 8.64; CV 0.636)
Calf survival rate		NA	88.33 (SD 6.67; CV 0.076)
Juvenile survival rate		NA	96.92 (SD 1.50; CV 0.015)
Adult survival rate		NA	98.43 (SD 1.02; 0.010)

^1^Dispersal rate for Shark Bay was derived from a genetic study (Krützen et al.2004a) (see Appendix S2).

^2^Shark Bay population size estimate was obtained from Preen et al. (1997).

^3^SD_EV_ (standard deviations due to environmental variance), and corresponding coefficients of variation (CV_EV_) are shown in brackets. Note that CVs for reproductive rates are consistently higher than for survival rates.

^4^Juvenile survival rates for the Shark Bay population were subdivided into ‘juve‐1’ (3–6 years), ‘juve‐2’ (6–9 years), and ‘subadults’; subadult categories for males range from age 9–15 years, but for females, who mature earlier, from age 9 to 12 years (see Table S2).

^5^Bunbury annual vital rates (not used as vortex input) are shown for comparison. Further details of parameter estimation methods are in Appendices S1 and S2.

We estimated input parameters for the Bunbury model from 3 years of field surveys (2007–2010). During this period, 212 transect surveys were conducted over 228 field days, throughout all seasons, sighting 578 groups of dolphins (Smith et al. 2013). For the Bunbury population, we estimated the population size based on photo‐identification surveys between February 2007 and March 2010 (Smith 2012; Smith et al. 2013). Unlike Smith et al. (2013) and Sprogis et al. (2016a), who reported seasonal abundances our estimates of population size and vital rates are for the entire three‐year survey period (2007–2010). We identified a total of 259 individuals of which 243 were known to be alive throughout this three‐year period. To account for any unmarked individuals that may have been missed in the census, we added an additional 10% to give an estimated population size of 267. The addition of 10% assumes that approximately 90% of the individuals in the population were accounted for. This value is derived from two other studies by our collaborators on coastal dolphins with high site fidelity and large survey effort. (1) The estimated percentage of distinctive individuals detected in three species of coastal dolphins at three different sites in Western Australia ranged between 89% and 100% (93–95% for bottlenose dolphins) (Brown et al. 2016). (2) In capture–mark–recapture analyses of the Bunbury population, Sprogis et al. (2016a) estimated similar marked percentages, ranging from 80% to 90%. The carrying capacity estimate for the Bunbury population was set at 370. This value was calculated by applying the ratio of SB carrying capacity/population size ratio (4000/2888) to the Bunbury population size.

The age classes were divided into three‐year intervals because this best reflects major changes in development (Connor et al. 2000; Mann et al. 2000), but also because there are not sufficient data on single‐year age classes to allow accurate estimates. We determined the distribution of the three main age classes: calves, juveniles, and adults (Table 1; Appendix S2).

As a result of following them for many years, we know the approximate date of birth for the majority of individuals in SB. Consequently, for the SB dolphins age classes could be confidently determined from dates of birth for calves and juveniles, because most animals were observed within 1 or 2 years of birth. Age‐class structure of the Bunbury population (2007–2010) was based primarily on known dates of birth, but also relied on body size and behavior (Smith 2012; Smith et al. 2013). Calves were defined as individuals that had not yet been weaned by their mothers (age 0–3 years; vortex age category 0). If the date of birth was unknown, calves were determined based on size (1–1.5 m) and swimming in consistent proximity in “infant position” under the peduncle and tail flukes of the mother (Smith 2012). Dolphins were considered juveniles once they had been weaned, and were no longer maintaining infant position, but were less than age 12 years for females (vortex age categories 1–3) or 15 years for males (vortex age categories 1–4). We defined animals as adults at a time point when they typically first bear offspring – at age 12 years (vortex age categories 4–10) for females (Mann et al. 2000) and age 15 years (vortex age categories 5–10) for males, based on approximate age of stable male alliance formation (Connor et al. 2000). If the exact age was unknown, juvenile and adult age classes were determined based on size, a commonly used indicator for bottlenose dolphin age classes (e.g., Hale et al. 2000; Mann et al. 2000) – Bunbury juveniles were classified as being approximately up to 2 m long, adults approximately 2.5 m; SB juveniles and adults are slightly smaller. Additionally, adult females in SB were aged based on the degree of ventral speckling, because it correlates with age, and speckling onset has been associated with sexual maturity (Krzyszczyk and Mann 2012). Little or no body speckling has been observed on Bunbury bottlenose dolphins.

The division of stages into three‐year age classes was also suitable because the vital rate estimates comprised data from four‐three‐year intervals (SB: 1988–1999) and one‐three‐year period (Bunbury: 2007–2010). To assess the validity of this three‐year model, we also constructed a one‐year model based on one‐year age classes, annual vital rates, and corresponding variances. The one‐year model projections, as well as the overall results of sensitivity analyses, mirrored those of the three‐year model (data not shown).

To determine the proportion of adult females reproducing per three‐year observation period, we used snapshot analyses, estimating the number of females breeding, as a percentage of all adult females sighted during that period (see Appendix S3). The snapshot analysis accounts for all individuals in the population at a given time period and assumes approximate constancy of environmental effects on demography. The percentage of adult females reproducing in SB was based on a subset of the population consisting of 43 (1988–1990), 64 (1991–1993), 70 (1994–1996), and 78 (1997–1999) adult females. The percentage of Bunbury adult females reproducing per three‐year observation period was derived from 81 adult females (2007–2010). Hereafter, we use the term “reproductive rate” to refer to the percentage of adult females breeding per three‐year observation period.

We estimated three‐year survival rates for the three main age classes (calves, juveniles, and adults) for both populations. Survival rates of the SB population were estimated from information available in the SB dolphin database (Table 1). Survival rates were calculated from 274 individuals in the Bunbury population (2007–2009) and an average of 346.83 individuals across four‐three‐year time periods in SB (1988–1999). Survival rates required for population models can rarely be calculated directly from the data, especially age‐specific survival rates. However, in our study we could infer deaths from the survey data for animals whose age (or age class) could be estimated. For some individuals, exact age of death could be inferred if approximate birth dates were available (i.e., they were first sighted with fetal fold lines); for others, we inferred death at a particular age class (see information on determining age classes above). For some recovered carcasses, age at death could also be estimated based on teeth morphology (Raudino unpubl. data). Adults and juveniles that had been confidently photo‐identified and been surveyed annually for many years were assumed to have died once they had not been sighted for at least 3 years. Calves under 3 years of age were presumed dead if there were two or more sightings of the mother without the calf, or when a carcass was recovered.

Juvenile survival rates for SB were further grouped into four age subcategories for vortex input: juve‐1 (age category 1, ages 3–6 years), juve‐2 (age category 2, ages 6–9 years), subadult‐1 (both sexes: age category 3, ages 9–12 years), and subadult‐2 (only males: age category 4, ages 12–15 years) (see Table 1, Table S2, Appendix S2). Note that, because males mature on average three years later than females, there are only three juvenile subcategories for SB females (see Table 1; Table S2). In contrast, for the Bunbury juveniles we estimated only one survival rate (males: age categories 1–4, ages 3–15; females: age categories 1–3, ages 3–12 years) without any juvenile subclasses because we were able to assign individuals to the three main age classes (calves, juveniles, and adults), but the exact ages of many individuals were unknown.

We also considered CMR (capture–mark–recapture) methodology to estimate survival rates, but found it to be less suitable for this comparative PVA because not all age classes of the Bunbury population are sufficiently marked; in particular, calves may lack distinctive dorsal fin markings and are thus not always individually identifiable (see Appendix S4). For that reason, we did not rely on CMR for the models. Nevertheless, we also evaluated the applicability of CMR methods and found that application of CMR‐derived survival rates (Nicholson et al. 2012; Smith et al. 2013) did not change the overall findings of our analysis (Appendix S4).

We calculated standard deviations due to environmental variance (SD_EV_) for reproductive and survival rates (see Appendix S3), and these variances were used to define the distributions of demographic rates from which values are sampled in each three‐year time period of the simulation model. Because the Bunbury data comprised only one‐three‐year time period, we adopted the long‐term variance from the SB survival rates. To estimate three‐year SD_EV_s for Bunbury survival rates, we calculated coefficients of variation for the SB mortality rates (*SB‐CV*_*EV*_ *= SB‐SD*_*EV*_ */mean mortality*) and applied them to the Bunbury rates (*three‐year Bunbury‐SD*_*EV*_ *= SB‐CV*_*EV*_ *x Bunbury‐mortality*). This was justified because the rank orders of Bunbury annual variances and coefficients of variation were identical to the corresponding three‐year SB rank orders – reproductive rates being the most variable, followed by calf survival, juvenile survival, and then adult survival (Table 1).

Genetic data for SB indicate mild differentiation between the eastern and western gulf (Krützen et al. 2004a), so the gulfs were modeled as two subpopulations connected by dispersal (Appendix S2). Our preliminary tests showed that the relatively small dispersal between the two subpopulations had very little effect on the model forecasts for the entire SB population. For details on how we calculated dispersal rates, see Appendix S2. Unlike Tsai and Mann (2012), we define dispersal as a measure of gene flow between the two gulfs. The Bunbury population was modeled as a single population because it has no physical boundaries, and there are no available data on dispersal. We also estimated sex ratios and the proportion of males contributing to the gene pool (Table 1; Appendix S2).

vortex simulates inbreeding depression as a decrease in first‐year age‐class survival among inbred individuals as a function of lethal equivalents (Miller and Lacy 2005). vortex calculates the inbreeding coefficient for each simulated individual from its pedigree, and then simulates inbreeding depression as a decrease in first‐year age‐class survival among inbred individuals. The survival rate of an inbred individual is specified in the model as *S *= *S*_0_ * exp (−*bF*), in which *S*_0_ is the survival rate for noninbred individuals, *F* is the inbreeding coefficient, and *b* is the “lethal equivalents” per haploid genome – a common measure of the severity of inbreeding depression (Miller and Lacy 2005). Thus, as some individuals within a small population become inbred due to mating between relatives, the mean survival rate for the population will decrease. Data on inbreeding (Frère et al. 2010) are complex and age‐specific, so we omitted inbreeding depression from the standard models, but evaluated its potential effect with sensitivity analyses by varying the number of lethal equivalents.

Note that vital rate and abundance estimation for both populations is an ongoing effort and previous reports include various methods and estimates for seasonal, site‐specific and sex‐specific abundance, and survival rate estimates for the two populations (e.g., Preen et al. 1997; Nicholson et al. 2012; Krzyszczyk 2013; Smith et al. 2013; Sprogis et al. 2016a). Therefore, future PVAs and other studies might update vital rates and abundance estimates for their specific purposes and aims, as more information becomes available.

### Standard models

To provide the most likely forecast for each population, we simulated standard models that were based on our best estimates for all input parameters. The main parameters for the standard models are given in Table 1. The standard models were run for 100 three‐year intervals to generate (maximum) 300‐year forecasts. Each scenario was repeated for 1000 iterations (replications) of the 300‐year projection with that set of input parameters. These standard models were then compared to scenarios that altered input values, as described below.

### Substituting input values

To test whether the difference in viability of the two populations is primarily caused by the differences in reproductive or survival rates, we substituted the respective parameter values. We applied the reproductive and survival rates of the Bunbury population to the SB model, and vice versa. This also allowed us to assess the effectiveness of wildlife management targets that are aimed at either increasing reproduction or survival. We also tested whether any difference in the viability of the two populations might be explained by the difference in population size, by substituting the respective initial population sizes (*N*_0_) and associated carrying capacities.

### Sensitivity analyses

We conducted fixed‐proportion and observed‐variation sensitivity analyses to evaluate the effect of potentially important parameters on population trajectories. Five input parameters were altered: (1) reproductive rates, (2) calf survival, (3) juvenile survival, (4) adult survival rates, and (5) the number of lethal equivalents. The standard model values served as the “medium” values for each analysis, except for inbreeding. For the medium value for inbreeding, we used 3.14 lethal equivalents, which is the median number of lethal equivalents reported for 38 mammalian species (Ralls et al. 1988). Alteration of the five parameters produced high, medium, and low input values.

#### Fixed‐proportion analyses

In the fixed‐proportion analyses, we varied the five input parameters by equal proportions of ± 1%, giving three input categories: high (+1%), medium (standard value; 3.14 lethal equivalents), and low (–1%).

#### Observed‐variation analyses

In the observed‐variation analyses, we varied the reproductive and survival rate standard values by ± 1 SD_EV_ (reflecting observed temporal variation of vital rates). The number of lethal equivalents was varied from 0 to 3.14 and 6.28. Thus, for each of the five parameters we generated three input values: high (+1 SD_EV_ for reproductive and survival rates; 6.28 for lethal equivalents), medium (standard value; 3.14 lethal equivalents), and low (–1 SD_EV_; 0 lethal equivalents).

For both types of analyses, we ran 243 scenarios to test all combinations (3^5^) of values of the five input parameters. We used these simulations to evaluate the sensitivity of stochastic growth rate (*r*) and 100‐year population size (*N*_100_) forecasts, in response to input parameter perturbations. Kruskal–Wallis, a nonparametric test, was used to compare median output values generated by the high, medium, and low input values. We ranked the relative effect of input parameters according to the Kruskal–Wallis *H*‐values, assigning the highest rank to the input parameter whose variation resulted in the largest fluctuations in output values. We also calculated elasticity values, which measure the relative contribution of each vital rate to population growth (de Kroon et al. 1986) (Appendix S6).

## Results

### Demographic comparison between populations

The SB population has a population size of approximately 2900 individuals – more than ten times larger than the Bunbury population (ca. 260 individuals). The biggest difference in vital rates between the two populations was reproductive output. The percentage of females breeding per three‐year period in SB ranged from 48.44% to 72.09% with a mean of 58.35% (Fig. S3A). The three‐year reproductive rate of the Bunbury population was 40.74%, which is lower than the lowest observed three‐year reproductive rate of the SB population (Fig. S3A; see Appendix S3 for statistical comparison). In contrast to reproductive rates, there was little difference in survival rates between the two populations (Table S3). Noticeably, there was no significant difference between SB and Bunbury noncalf (i.e., juvenile and adult combined) survival rates, which is consistent with data derived from the use of CMR methodology that show identical noncalf survival rates of 95.0% for both populations (Nicholson et al. 2012; Smith et al. 2013) (see Table S3). Among all vital rates, reproductive rates displayed the largest observed, temporal variation (Table 1; Fig. S3). In contrast, survival rates were relatively constant over time (Table 1; Fig. S3).

### Standard models

**Figure 1:**
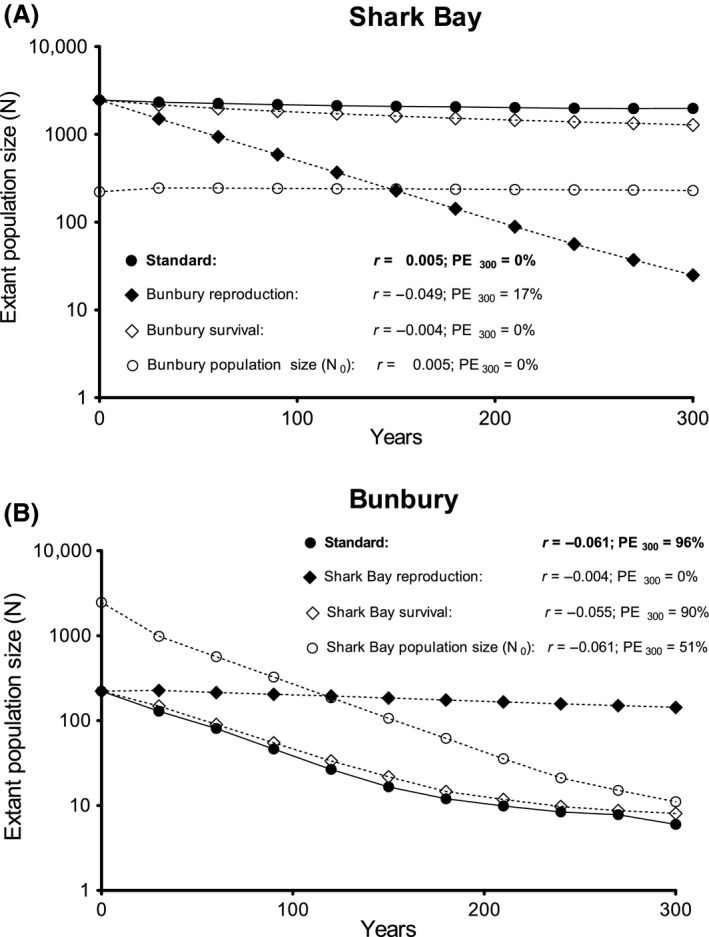
Population trajectories of standard models; effect of substituting survival rates, reproductive rates and initial population sizes (*N*_0_) on population trajectories, stochastic growth rates (*r*), and forecasts for 300‐year probabilities of extinction (*PE*_300_). (A) Effect of application of Bunbury vital rates and *N*_0_ to Shark Bay standard model. (B) Effect of application of Shark Bay vital rates and *N*_0_ to Bunbury standard model. Values for extant population size forecasts were log‐transformed for better visual comparison of models with large differences in *N*_0_. Note that standard errors are too small to be shown on the graph – see Table S8 (Appendix S8) for standard errors for each population size forecast plotted here.

The SB model forecast a stable population. The population trajectory of the SB model displayed a very small positive stochastic growth rate (*r*) of 0.005 (Fig. 1A; Table S5). The population size of the SB population was forecast to tally 2162 (SE 10) individuals after 100 years (*N*_100_) and 1980 (SE 12) after 300 years (*N*_300_). The probability of extinction after 300 years (*PE*_300_) was 0.0% (Table S5). Note that the forecast population size trajectory of the SB population shows a slight decline (Fig. 1A), despite the fact that its growth rate is slightly positive. This is because in vortex the growth rate is calculated before carrying capacity is imposed, so that the average population size that is impacted by carrying capacity limitations on growth will be depressed relative to simulations without carrying capacity limitations.

In contrast to SB, the Bunbury standard model forecasts a declining population (*r* = −0.061) with a rapidly increasing extinction risk after about 150 years (Fig. 1B; Table S5). Not including potential migrants, Bunbury was forecasted to have 38 (SE 0.6) dolphins after 100 years – an 83% decline. After 300 years, there were forecasted to be 6 (SE 0.6) individuals, with a 96% probability of extinction. The mean time for extinction was 203 (SE 1.32) years (Table S5).

### Substituting input values

Substituting reproductive rates had a much greater influence on population viability than substituting survival rates or initial population sizes (Fig. 1). Applying the reproductive rate (40.74%) of the Bunbury population to the SB model caused the otherwise stable SB population to sharply decline to 25 individuals over a 300‐year period (*r* = −0.049) (Fig. 1A). Conversely, applying the SB mean reproductive rate (58.35%) to the Bunbury model, the Bunbury population approached stability, raising r by 93% (from *r *= −0.061 to *r *= −0.004), and lowering *PE*_300_ from 96% to 0% (Fig. 1B). In contrast to the results for reproduction, substituting survival rates had relatively little effect. Application of the Bunbury survival rates to the SB model slightly lowered the population trajectory (from *r *=* *0.005 to *r* = −0.004), but did not raise the probability of extinction (*PE*_300_ = 0%) (Fig. 1A). Applying the SB survival rates to the Bunbury model elevated *r* by less than 10% (from *r *= −0.061 to *r* = −0.055), maintaining a large *PE*_300_ of 90% (Fig. 1B). Substituting the initial population sizes had no effect on growth rate forecasts and a much lesser effect on *PE*_300_‐ and *N*_300_‐forecasts than substituting reproductive rates (Fig. 1).

### Sensitivity analyses

#### Fixed‐proportion analyses

**Figure 2:**
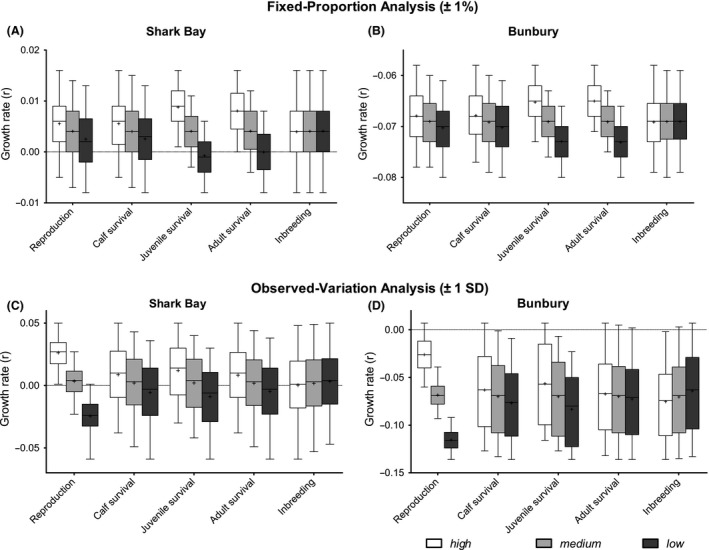
Sensitivity analyses. Panels A, B: relative effect of fixed‐proportion perturbations (standard value ± 1%; 3.14 lethal equivalents ± 1%) of parameters on stochastic growth rate (*r*). Panels C, D: relative effect of observed, temporal variation in reproductive rates and survival rates (standard value, ±1 SD_EV_), as well as perturbations of inbreeding levels (0, 3.14, 6.28 lethal equivalents) on stochastic growth rate (*r*) forecasts. Each box plot shows median, upper, and lower quartile growth rate forecasts of 81 simulations across all (3^4^) combinations. The white, gray, and dark‐shaded boxes show the output from scenarios run with *high*,* medium,* and *low* input values, respectively. Whiskers display minimum and maximum output value.

The effects of proportional alterations (±1%) of the input parameters – reproductive rates, calf, juvenile, adult survival rates, and inbreeding – on population growth and population size were ranked according to Kruskal–Wallis *H*‐values (Table 2). Inbreeding had a very small and nonsignificant effect in all cases (Fig. 2; Table 2) and will not be discussed further. For both populations, growth rate and *N*_100_ were most sensitive to proportional changes in adult and juvenile survival rates (Figs. 2A and B, S7A and B; Table 2). Proportional changes of reproductive rates had relatively little effect on *r* (Fig. 2A and B; Table 2) and *N*_100_‐forecasts (Fig. S7A and B). The rankings based on elasticity values were concordant with the rankings based on Kruskal–Wallis *H*‐values for the fixed‐proportion analysis (Table S6).

**Table 2:** Observed‐variation and fixed‐proportion sensitivity analyses: Effects of parameter variation on stochastic growth rate (*r*) and 100‐year population size (*N*_100_) forecasts for the Shark Bay and Bunbury population

			Growth rate (*r*)						Population size (*N*_100_)					
			High	Medium	Low	H (*KW*)^1^	Sig.^2^	Rank	High	Medium	Low	H (*KW*)^1^	Sig.^2^	Rank
Shark Bay	Observed‐variation Analysis	Reproduction	0.0262	0.0034	−0.0245	189.3	****	1	2694	2091	1175	187.2	****	1
		Calf survival	0.0088	0.0020	−0.0055	12.8	**	3	2171	1990	1798	11.6	**	3
		Juvenile survival	0.0121	0.0020	−0.0089	27.5	****	2	2277	1995	1686	30.2	****	2
		Adult survival	0.0082	0.0018	−0.0048	10.2	**	4	2168	1988	1805	10.6	**	4
		Inbreeding	0.0003	0.0017	−0.0032	0.5	ns	5	2179	1984	1996	0.0	ns	5
	Fixed‐proportion Analysis	Reproduction	0.0055	0.0040	0.0025	11.4	**	3	2183	2132	2074	10.5	**	3
		Calf survival	0.0055	0.0040	0.0025	11.1	**	4	2183	2129	2077	10.0	**	4
		Juvenile survival	0.0088	0.0041	−0.0008	126.2	****	1	2310	2130	1949	133.0	****	1
		Adult survival	0.0080	0.0041	0.0000	87.2	****	2	2275	2127	1987	77.6	****	2
		Inbreeding	0.0040	0.0041	0.0041	0.0	ns	5	2129	2130	2130	0.0	ns	5
Bunbury	Observed‐variation Analysis	Reproduction	−0.0261	−0.0684	−0.1155	211.0	***	1	145	39	8	213.6	***	1
		Calf survival	−0.0631	−0.0700	−0.0768	5.1	ns	3	77	63	51	5.2	ns	3
		Juvenile survival	−0.0565	−0.0701	−0.0834	21.0	***	2	90	62	39	21.3	***	2
		Adult survival	−0.0673	−0.0700	−0.0727	0.8	ns	5	69	63	59	0.9	ns	4
		Inbreeding	−0.0754	−0.0704	−0.0642	3.4	ns	4	67	63	61	0.5	ns	5
	Fixed‐proportion Analysis	Reproduction	−0.0679	−0.0690	−0.0703	8.7	*	4	37	35	34	9.8	**	3
		Calf survival	−0.0679	−0.0692	−0.0702	9.0	*	3	37	35	34	9.1	*	4
		Juvenile survival	−0.0652	−0.0691	−0.0730	103.1	****	2	41	35	30	107.7	****	2
		Adult survival	−0.0650	−0.0691	−0.0731	114.5	****	1	41	35	31	108.6	****	1
		Inbreeding	−0.0691	−0.0691	−0.0691	0.0	ns	5	35	35	35	0.0	ns	5

“High,” “medium,” and “low” refer to manipulations of input variables described in text.

^1^Kruskal–Wallis *H*‐values were used to rank relative effect of the five input parameters on r‐ and N_100_‐forecasts.

^2^Significance levels (sig.) indicate significant differences between the output from scenarios with high, medium and low input values according to Kruskal–Wallis tests: ns, not significant; *P < 0.05; **P < 0.01; ***P < 0.001; ****P < 0.0001.

#### Observed‐variation analyses

In contrast to the fixed‐proportion analyses, the observed‐variation analyses showed that population viability was most affected by variation in reproductive rates, followed by juvenile survival, but not strongly by adult survival (Figs. 2C and D, S7C and D; Table 2). Reproductive and juvenile survival rates were the only variables which consistently gave significant results (Table 2). For instance, an increase or decrease in SB reproductive rates by one SD_EV_ corresponded to a 671% increase (from *r* = 0.0034 to *r *=* *0.0262) or an 821% decrease (from *r *= 0.0034 to *r *= −0.0245) in *r*, respectively. The mean SB population size forecast after 300 years (*N*_300_) was 385 (SE 43) for all 81 simulations with low observed variation in reproductive rates (− 1 SD_EV_) and 2637 (SE 32) for all high reproductive rate combinations (+1 SD_EV_). Observed temporal variation in adult survival rates had the least influence on population dynamics and did not significantly alter *r*‐ and *N*_100_‐projections for the Bunbury population (Figs. 2C and D, S7C and D; Table 2). Also, the mean SB population size forecasts after 300 years (*N*_300_) were relatively insensitive to observed variation (±1 SD_EV_) in adult survival, varying between 1352 (SE 115) for − 1 SD_EV_ and 1818 (SE 112) for + SD_EV_. Moreover, altering reproductive rates by one SD_EV_ had a greater effect on population dynamics of both populations than changing survival rates of all age classes simultaneously (Table S9; Fig. S9).

## Discussion

For animals with slow population growth, adult survival has been considered to be more important than reproduction because, when changed at equal proportions, adult survival has a greater influence on population growth than reproduction (Brault and Caswell 1993; Heppell et al. 2000; Saether and Bakke 2000; Crone 2001; Oli and Dobson 2003; van de Kerk et al. 2013). Although our fixed‐proportion analysis does not contradict this, our study contributes to a growing body of work, which suggests that, if parameter values vary over the range observed, reproduction can be more important for population viability of slow‐growing populations than survival. Other studies show that this is true for other slow‐growing vertebrate populations (e.g., Beissinger and Peery 2007; Mitchell et al. 2009; Johnson et al. 2010).

The Bunbury population was projected to decline and is at risk of extinction, unless supported by immigration. In contrast, the SB population, which is exposed to relatively little human activity, appears to be stable (Fig. 1; Table S5). This difference in viability raises important questions: What makes one population decline and another stable? What does this tell us about the relative importance of reproduction and survival? Four findings (1–4 below) indicate that reproduction may be considerably more important for conservation than results of the commonly used, fixed‐proportion analyses would suggest:

1. **Reproductive rates were higher and adult survival rates lower in the stable population**. Compared to the apparently declining Bunbury population, reproductive rates were higher, and adult survival rates were lower in the stable SB population (Table 1; Fig. S3).
2. **The difference in viability is best explained by reproduction.** The difference in viability of the two dolphin populations was primarily due to the difference in reproductive rates. Substitution of reproductive rates had the greatest effect on model forecasts (Fig. 1). On the other hand, survival contributed little to the difference in viability of the two populations – substitution of survival rates had only a very small effect on the forecast of either model (Fig. 1).
3. **Reproductive rates are variable; survival rates are relatively constant.** Our long‐term data on the SB population showed that reproductive rates displayed large temporal fluctuations, whereas survival rates were relatively constant over time (Table 1; Fig. S3).
4. **Natural variation in reproduction had the greatest influence on population viability.** The larger variation in reproductive rates also resulted in reproduction having a larger impact on population viability as measured by the observed‐variation analysis (Fig. 2C and D; Table 2).

Our finding (3) that reproductive rates are variable whereas survival rates are relatively constant has been reported in several other studies on slow‐growing taxa. This includes observations on elk (*Cervus elaphus*) (Raithel et al. 2007), deer and sheep species (Gaillard et al. 1998), grizzly (*Ursus arctos*) (Harris et al. 2006), black bears (*Ursus americanus*) (Mitchell et al. 2009), and snow geese (Cooch et al. 2001), which all show high variation in recruitment, but low variation in (adult) survival.

Our finding (4) that the larger temporal fluctuations in reproduction also translate to reproduction having a greater influence on population viability has been addressed in several studies. For instance, a study on black bears showed that, although growth rates were most sensitive to proportional changes in juvenile and adult survival, long‐term fluctuations in reproductive rates had a greater impact on growth rates than survival (Mitchell et al. 2009). Similar findings were reported for several other animal populations, including mallards (*Anas platyrhynchos*) (Hoekman et al. 2002), cowbirds (*Molothrus ater*) (Citta and Mills 1999), bighorn sheep (*Ovis canadensis sierrae*) (Johnson et al. 2010), and sage grouse (*Centrocercus urophasianus*) (Taylor et al. 2012). For example, in a meta‐analysis of 86 black bear populations, it was shown that the eastern North American populations displayed higher reproductive rates and lower adult survival than their less stable western counterparts. The difference in growth rates, was best explained by differences in reproduction (Beston 2011). A Population decline (1969–1990) of snow geese (*Chen caerulescens*) was primarily due to the simultaneous decrease in reproduction (Cooch et al. 2001), despite the fact that growth rates of these birds were shown to be most sensitive to proportional changes in adult survival (Rockwell et al. 1997). Likewise, observed, temporal variation in reproduction, and not survival, was shown to be the greatest contributor to projected population declines in the marbled murrelet population mentioned in the Introduction section (Beissinger and Peery 2007).

### Model limitations

Like all models, the forecast of our PVA model is limited by the model assumptions and accuracy of input parameters. As outlined in the methods, our estimates for survival rate estimates, as well as population size estimates, are survey‐based estimates. In other words, we did not apply CMR analyses that are commonly used to estimate demographic parameters of dolphin populations (e.g., Nicholson et al. 2012; Smith et al. 2013; Sprogis et al. 2016a). Therefore, we believe these two parameters deserve further discussion. Our survey‐based survival estimation of survival rates and population size of the BB population was tenable due to the large survey effort and high site fidelity. For example, in Bunbury one male was sighted in 2007, not in 2008, and was sighted again in 2009. All other individuals in that population were sighted at least annually – including adult males who temporarily depart from the survey site, but return seasonally for mating opportunities (Smith et al. 2013; Sprogis et al. 2016a,b). Likewise, SB bottlenose dolphins of both sexes show high site fidelity (Tsai and Mann 2012).

#### Survival rate estimates

As detailed in the methods, for our standard models we did not use CMR‐derived survival rates because it was not possible to use CMR to reliably estimate calf survival rates of both populations. The reason for this is that calves are – at least for the Bunbury population – not yet sufficiently marked, that is, they lack distinctive dorsal fin markings (H. Smith & K. Nicholson, pers. comm.). Given that PVA modeling requires survival rates for all age classes, calf survival cannot be neglected, especially because calf survival rates are typically much lower than those of noncalves. Therefore, in order to compare population dynamics of the two populations – which requires using the same reliable methodology for both populations – we did not use CMR‐derived survival rates for the standard models.

Furthermore, we showed that CMR‐based noncalf survival rate estimates (from Nicholson et al. 2012 for SB; Smith et al. 2013 for Bunbury) did not alter the overall findings of our forecasts compared to our standard models that used survey‐based survival estimates. In comparison with the standard models, using survival rates estimated with CMR methods resulted in slightly different forecasts, but did not change the overall findings (see Appendix S4, Fig. S4). Regardless of which survival rates we used – CMR‐based or the survey‐based survival rates of the standard model – the scenarios all showed a big difference in viability of the two populations (Fig. S4). This was also the case when using noncalf survival rate estimates based on a 25‐year CMR analysis of the SB population (unpubl. data; Krzyszczyk 2013; E. Krzyszczyk, pers. comm.).

#### Population size estimates

For the SB population, we used the population size estimate based on aerial surveys by Preen et al. (1997). For the Bunbury population, we estimated population size based on photo‐identification surveys and adjusted this census count on the basis of the proportion of marked individuals detected in CMR studies (Brown et al. 2016; Sprogis et al. 2016a). A full CMR analysis to estimate the Bunbury population size might improve accuracy of this parameter and thus the PVA forecast. However, given the large survey effort and high site fidelity of the dolphins, we are confident that our adjusted census count is close to the actual population size. Furthermore, by substituting population size estimates between the SB model and the Bunbury model, we showed that population size estimates had very little effect on the forecast population trajectories (Fig. 1).

Finally, we would like to note that vital rate and abundance estimation for both populations is an ongoing effort and previous reports include various methods and estimates for seasonal, site‐specific and sex‐specific abundance, and survival rate estimates for the two populations (e.g., Preen et al. 1997; Nicholson et al. 2012; Krzyszczyk 2013; Smith et al. 2013; Sprogis et al. 2016a). Therefore, future PVAs and other studies might update vital rates and abundance estimates for their specific purposes and aims, as more information becomes available.

Another limitation with respect to the Bunbury forecast is related to the three‐year sampling period. Given that we had only one‐three‐year sampling window for Bunbury, and because reproductive rates of the SB population displayed large temporal fluctuations, the relatively low reproductive rate observed for Bunbury might have been an outlier. We thus encourage continuous monitoring of the Bunbury population demographic parameters, especially reproductive rates. Nevertheless, if this low reproductive output persists over a longer time period, this would be reason for concern.

### Management implications

**Figure 3:**
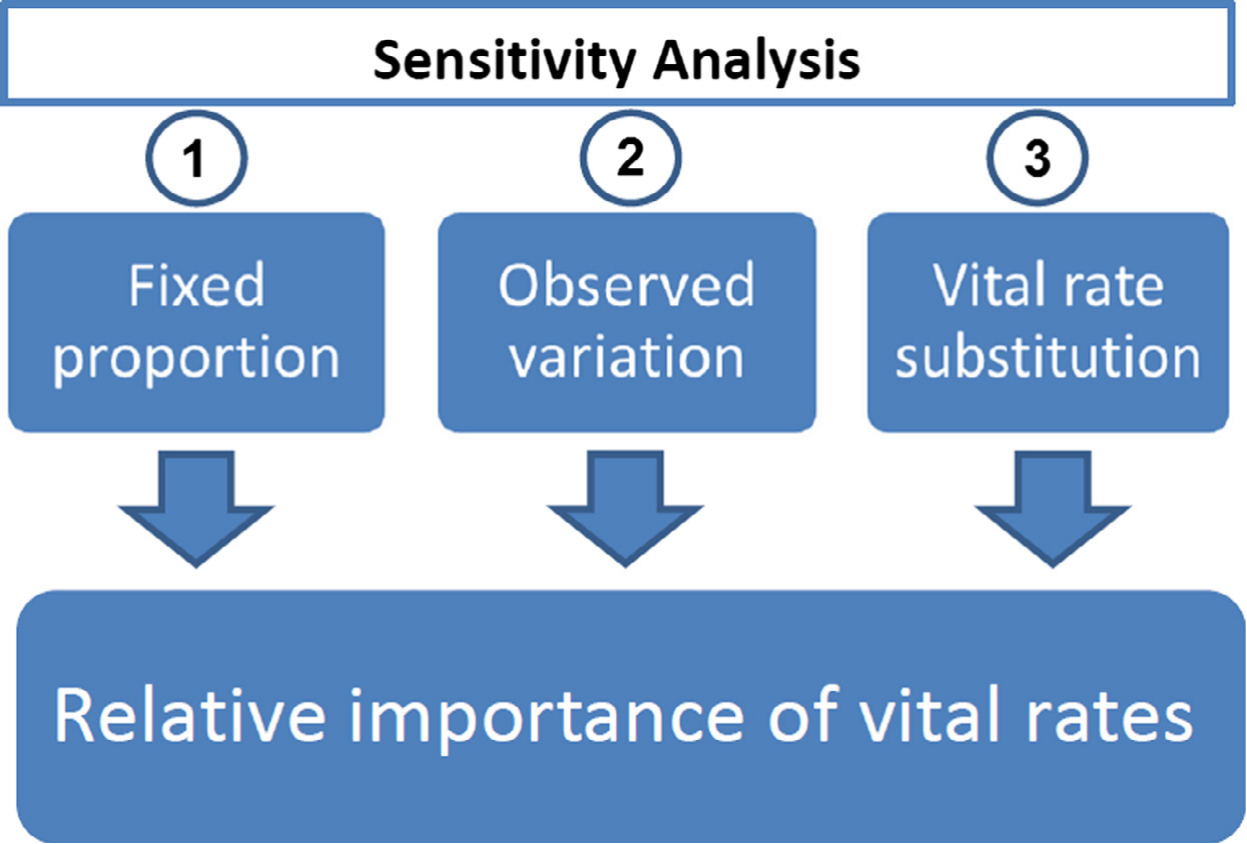
Approach to assessing the importance of vital rates for the conservation of the two bottlenose dolphin populations. We combined (1) fixed‐proportion and (2) observed‐variation sensitivity analyses with (3) methods of vital rate substitutions between contrasting populations and threat assessment. This approach provided insight into the relative importance of reproduction and survival for the two dolphin populations, and guidance to wildlife management.

The results of the observed‐variation analysis apparently contradict the findings of the commonly used fixed‐proportion analysis. The fixed‐proportion analysis suggests that factors affecting survival are most important, whereas the observed‐variation analysis suggests that reproduction has the greatest impact on population viability. This discrepancy is commonly explained by natural differences in plasticity of vital rates: demographic parameters that have large effects on growth rates, as measured by fixed‐proportion analyses, tend to display low levels of natural fluctuations, possibly due to canalization of a given rate (Gaillard et al. 1998, 2000; Pfister 1998; Mills and Lindberg 2002; Gaillard and Yoccoz 2003; Raithel et al. 2007; Schmutz 2009). However, wildlife managers usually need to act on factors that alter vital rates on a proximate timescale – rather than an evolutionary timescale. In comparison with fixed‐proportion analyses, analyses that incorporate observed variability may better reflect the effect of changes on vital rates on a proximate timescale. Consequently, conservation efforts should focus on vital rates that have an effect on a proximate timescale, and can be impacted by management, rather than focusing on generalizations derived from infinitesimal analytically based elasticity and sensitivity measures. To assess the importance of vital rates for conservation of the two dolphin populations, we used multiple approaches (see Fig. 3). In addition to the commonly used fixed‐proportion analysis (1), we assessed the effect of observed vital rate variation on population dynamics (2), compared and substituted vital rates between the two contrasting populations (3). This allowed us to assess to which rates are depressed relative to stable populations, which in turn gave us clues to potential threats (Fig. 3). This approach offered insight into feasibility and effectiveness of wildlife management options.

#### Feasibility of wildlife management options

The importance of vital rates for conservation is heavily dependent on the feasibility of management actions, that is, the degree to which each vital rate can be manipulated by wildlife managers. Vital rates rarely change by similar proportions in nature (Mills et al. 1999; Mills 2007), and management actions may not succeed in altering vital rates over the range typically used in proportional perturbations (de Kroon et al. 2000). For instance, raising reproductive rates of the Bunbury population by 5% (from 40.74% to 42.78%) seems feasible – such an increase is well within the range of natural fluctuations observed in SB. In contrast, boosting adult survival rates by the same proportion (from 95.95% to 100.7%) is impossible. Thus, even if slow‐growing populations tend to be most sensitive to proportional changes in adult survival, it may be much harder to increase survival rates that naturally display little variation. Managers, therefore, would benefit from using PVAs that incorporate achievable changes in vital rates and that reflect variability likely to occur in wild populations.

#### Effectiveness of wildlife management options

From a conservation perspective, the importance of vital rates also depends on the effectiveness of management actions aimed at altering them. This in turn also depends on current and future levels of vital rates, as well as factors that alter them. A population that is declining as a result of low levels of one vital rate is unlikely to be rescued by attempting to increase a different vital rate that is currently at a level that approaches the maximum attainable for the species. For instance, it is possible to prevent the forecast population decline of the Bunbury population by raising reproductive rates, but increasing survival rates is relatively ineffective (Fig. 1B) because these rates are already comparatively high. Therefore, the rule of thumb proposed by van de Kerk et al. (2013) – that for slow‐growing animal populations strategies targeted on adult survival are expected to be more effective – may need reconsideration.

#### Importance of vital rates is context‐ and population‐specific

The difference in viability between the SB population and the less stable Bunbury population is due to reproduction (Fig. 1). Consequently, in this case, focusing management efforts on raising the relatively low reproductive output of the Bunbury population would be the best option. This may be different for other populations, such as the offshore bottlenose dolphins in the Pilbara region in Western Australia, which experience greater levels and fluctuations in mortality due to bycatch (Allen et al. 2014). Likewise, there is an indication that an observed decline in local abundance is associated with unusually high calf mortality in bottlenose dolphins off New Zealand (Tezanos‐Pinto et al. 2015). Similarly, population growth of black bears has been shown to be most affected by changes in reproductive rates in protected areas, where adult survival is relatively high and constant. On the other hand, in unprotected areas, with larger fluctuations in adult survival rates due to hunting, variation in adult survival had a greater influence on population growth (Mitchell et al. 2009).

This example of black bears in protected versus unprotected areas (Mitchell et al. 2009) illustrates that the variability and thus importance of reproduction versus survival is context‐ or population‐specific. Similarly, another study found large spatial and temporal variation in vital rates of bighorn sheep populations, which differed in their importance in different populations (Johnson et al. 2010). This population‐specific variation in the importance of vital rates has been shown in other slow‐growing populations of the order Artiodactyla (e.g., Albon et al. 2000; Coulson et al. 2005; Raithel et al. 2007; Nilsen et al. 2009). Consequently, Johnson et al. (2010) concluded that “such shifts in the means and variances of key vital rates may be largely responsible for declining and endangered populations” (p. 1763). Therefore, identifying which vital rates are depressed below observed levels and fluctuations relative to stable populations may better inform wildlife management decisions, than relying on fixed‐proportion analyses or generalizations based on shared life histories.

#### Wildlife management options

Our results also show that, in comparisons with efforts to increase survival, management strategies that are aimed at increasing reproductive rates are likely to be more feasible and more effective at reversing the forecast decline of the Bunbury population. Therefore, in this case, reproduction should be the main focus of management actions. However, it should be noted that, although the Bunbury population can approach stability if reproductive rates are increased to the level of the SB reproductive rates, even at those levels the forecast growth rate was slightly negative (Fig. 1B). Therefore, in order to optimize population recovery, the best long‐term strategy would target both reproduction and survival, especially juvenile survival, which was lower for Bunbury and ranked second in all sensitivity analyses (Table 1; Table 2).

Actions aimed at reversing or preventing population declines often address both survival and reproduction, rather than just one vital rate at a time. To some degree, this is also true for the two dolphin populations, but some potential management actions have a greater effect on either reproduction or survival. In order to identify such management options, it is necessary to consider which factors influence vital rates and the viability of coastal dolphin populations. It has been shown that the presence of boats causes both short‐term behavioral disruptions (Lusseau 2005; Bejder et al. 2006a) and can lead to a long‐term decline in relative abundance of coastal dolphins (Bejder et al. 2006b). Specifically, reproduction has been linked to boat presence (Bejder 2005; Lusseau et al. 2006), but it should also be noted that in some cases “the dolphins are able to compensate for their immediate behavioural response to disturbances by commercial vessels” (New et al. 2013, p. 314). Still, it is feasible that the greater vessel traffic in Bunbury waters compared to Shark Bay might have contributed to the relatively low reproductive rates observed for the Bunbury population.

What wildlife management options are there to address this? In marine conservation, implementation of protected areas, including time closures and area closures, is an effective management strategy (Edgar et al. 2014; Tyne et al. 2015). Vessel restrictions, in particular speed limits, could lower the incidents of vessel collisions and dolphin mortality of all age classes (Smith et al. 2016). However, our data show that for the Bunbury population it is important to target reproduction. In line with our findings, Smith et al. (2016) suggested that restrictions on vessel traffic, such as vessel exclusion zones and speed limits to prevent disturbances, could have a positive effect on mating behavior, reproduction, and ultimately the viability of the Bunbury population. This management option could specifically target reproduction when implemented during the peak in mating season for this population (Smith et al. 2013, 2016). Given that the peak mating and calving period coincide in bottlenose dolphins due to the 12‐month gestation of this species (e.g., Perrin and Reilly 1984; Schroeder 1990), restricting vessel traffic during that season could also have a positive effect on calf survival. Such management actions would ideally protect critical habitat (Hoyt 2011; Sprogis et al. 2016b). This could have a positive effect on reproductive success, if the areas and time periods targeted are important for socializing, including mating, calving, and nursing (Brough et al. 2016; Smith et al. 2016). This is analogous to resting areas that have been shown to be critical in Spinner dolphins (*Stenella longirostris*) off Hawai'i (Tyne et al. 2015).

Therefore, in the case of the Bunbury population, a specific vital rate, such as reproduction or calf survival, can be targeted to a certain degree, and our results indicate that these are appropriate vital rates to manage. While in the case of the two dolphin populations results are quite convincing, due to strong differences in reproductive rates, there are cases for which trends are less clear. In other species or populations, management actions may have multiple effects on vital rates. Also, in other species or populations, it would be best to perform a study such as ours to determine which vital rates are suitable targets for managements.

## Conclusion

Our comparative analysis of one stable and one apparently declining population provided valuable insight for both population biology and wildlife conservation. Several studies (e.g., Mills et al. 1999; Mills and Lindberg 2002; Morris and Doak 2002; Mills 2013) have previously identified that proportional sensitivity analyses may not always be the most useful analytical framework for conservation because they often fail to measure what is relevant in the application of PVA to conservation. However, despite this drawback, fixed‐proportion analyses are still commonly recommended and used without careful evaluation as to whether they meet the need. Likewise, generalizations on the basis of fixed‐proportion analyses persist, suggesting that (adult) survival is generally most important for the viability of slow‐growing populations (e.g., van de Kerk et al. 2013). Our findings support previous studies that challenge these generalizations (e.g., Mills et al. 1999; Mills and Lindberg 2002; Morris and Doak 2002; Mills 2013) by showing that, under realistic variation of parameter values (i.e., within the range of observed fluctuations), reproduction is much more important than results of proportional sensitivity and elasticity analyses would suggest. Moreover, the findings of this and other case studies may differ for different taxa, populations and context, that is, threats and natural variability. Therefore, we recommend that conservation‐oriented PVAs should assess the effect of alterations of vital rates that are likely to occur in wild populations, and whether those alterations are feasible and effective under alternative management scenarios.

## Conflict of Interest

None of the authors declare a conflict of interest.

## Supporting information


**Appendix S1** Study sites; *includes Fig. S1*
**Appendix S2** Parameters other than reproductive and survival rates; *includes Table S2*
**Appendix S3** Reproductive and survival rates; *includes Fig. S3*,* Table S3*
**Appendix S4** Applicability of capture‐mark‐recapture methodology; *includes Fig. S4*
**Appendix S5** Results of standard models: *Fig. S5*
**Appendix S6** Elasticity analysis; *includes Table S6*
**Appendix S7** Sensitivity analyses—effect on population size (*N*_100_); *includes Fig. S7*
**Appendix S8** Population size forecasts with associated standard error for Shark Bay standard, Bunbury standard and forecasts based on scenarios with substituted vital rates: *Table S8*
**Appendix S9** Effect of varying reproductive rates versus varying all age‐specific survival rates (±1 SD_EV_ **)** on population trajectories; *includes Table S9 and Fig. S9*
