# Supplementary material for: The relative importance of reproduction and survival for the conservation of two dolphin populations
Source: Ecol Evol. 2016 Apr 20;6(11):3496–512. doi: 10.1002/ece3.2130 (PMC5513288; doi:10.1002/ece3.2130)
Supplement: Supplementary file 1 — Appendix S1 Study sites; includes Fig. S1 Appendix S2 Parameters other than reproductive and survival rates; includes Table S2 Appendix S3 Reproductive and survival rates; includes Fig. S3, Table S3 Appendix S4 Applicability of capture‐mark‐recapture methodology; includes Fig. S4 Appendix S5 Results of standard models: Fig. S5 Appendix S6 Elasticity analysis; includes Table S6 Appendix S7 Sensitivity analyses—effect on population size (N 100); includes Fig. S7 Appendix S8 Population size forecasts with associated standard error for Shark Bay standard, Bunbury standard and forecasts based on scenarios with substituted vital rates: Table S8 Appendix S9 Effect of varying reproductive rates versus varying all age‐specific survival rates (±1 SDEV ) on population trajectories; includes Table S9 and Fig. S9 [file ECE3-6-3496-s001.docx]

**Appendix (Supplementary Material: S1-S9)**

**The relative importance of reproduction and survival for the conservation of two dolphin populations**

Oliver Manlik, Jane A. McDonald, Janet Mann, Holly C. Raudino^‡^, Lars Bejder, Michael Krützen, Richard C. Connor, Michael R. Heithaus, Robert C. Lacy and William B. Sherwin

^‡^ *nee* Smith

**S1** Study sites (Shark Bay & Bunbury); *includes Fig. S1*.

**S2** Parameters other than reproductive and survival rates; *includes Table S2*

**S3** Reproductive and survival rates; *includes Fig. S3*, *Table S3*

**S4** Applicability of capture-mark-recapture methodology; *includes Fig. S4*

**S5** Results of standard models: *Fig. S5*

**S6** Elasticity analysis; *includes Table S6.*

**S7** Fixed-proportion and observed-variation analysis—effect on population size forecasts (*N_100_*); *includes Fig. S7*.

**S8** Population size forecasts with associated standard error for Shark Bay standard,

Bunbury standard and forecasts based on scenarios with substituted vital rates: *Table S8*

**S9** Effect of varying reproductive rates versus varying all age-specific survival rates

(± 1 SD_EV_**)** on population trajectories; *includes Table S9 and Fig. S9*.

**S1 Study sites (Shark Bay and Bunbury)**

The two bottlenose dolphin (*Tursiops* cf*. aduncus*) populations that are the focus of our study are found in Shark Bay (SB) and in Bunbury coastal waters, Western Australia (Fig. S1). Shark Bay is listed as a World Heritage site in recognition of its outstanding natural values (Department of Arts, Sports, the Environment, Tourism and Territories, 1990; Department of Environment, Sport and Tourism 1995).The area supports the largest seagrass meadows in the world (Walker & Prince 1987). The bay is about 13000 km^2^ in size and is divided by the Peron Peninsula, which bisects it into a western and an eastern gulf. Approximately 2900 bottlenose dolphins inhabit the two gulfs of Shark Bay (Preen *et al*. 1997). Monkey Mia, situated on the eastern coast of the Peron Peninsula (Fig.S1a) is renowned for the interaction between humans and a very small proportion (< 0.2%) of the dolphins from that population (Mann & Kemps 2003; Foroughirad & Mann 2013). Vital rates on the Shark Bay population came from a subset of dolphins within a 300 km^2^ area in the eastern gulf of Shark Bay (see circled area in Fig. S1a).

The Bunbury study site encompasses about 120 km^2^ (Fig. S1b). Approximately 250 bottlenose dolphins use these waters around Bunbury (Smith *et al*. 2013). The dolphins are a major tourist attraction and represent a major contribution to the local economy. However, the Bunbury dolphin population faces increasing threats due to accelerating human disturbances. The regional city centre of Bunbury, with approximately 68000 residents (2011), has grown by more than seven per cent between 2009 and 2010 alone (Wahlquist 2011). Its port is one of the busiest in Australia, and the number of commercial vessels visiting the port has increased by over 33% since 2004 to a record number of 414 in 2011 (Bunbury Port Authority 2011). Coastal development and increased vessel traffic, including recreational vessels (Arcangeli & Crosti 2009; Jensen *et al*. 2009) are just some of the potential threats to the Bunbury population.

**
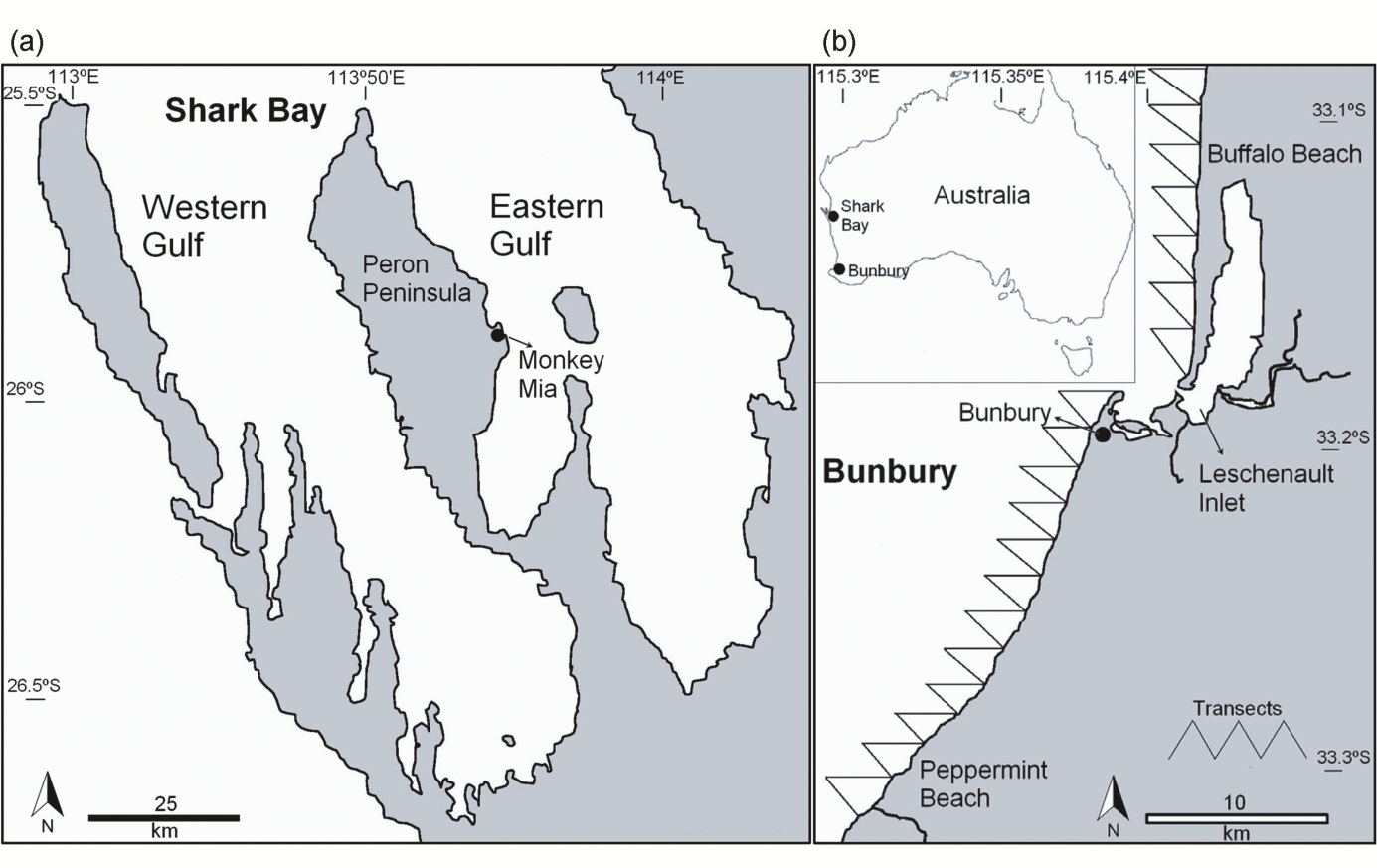
**

**Fig. S1.** (a) Shark Bay, a world heritage site, is about 13000 km^2^ in size and is divided by the Peron Peninsula, which bisects the bay into a western and an eastern gulf. Although whole of Shark Bay was modelled, the vital rates came from individuals in a 300 km^2^ area (circled) north of Monkey Mia. (b) The inset shows the relative location of the study sites (Shark Bay & Bunbury). The coastal study area of Bunbury covers about120 km^2^ and extends approximately 1.5 km offshore with a linear distance of 50 km. The study site includes the coastal areas, embayment, Leschenault Inlet and outer harbours (5 km^2^), estuary and river mouth (15 km^2^). Transects of the outer-water Bunbury study site are shown.

**References for S1**

Arcangeli, A. & Crosti, R. (2009) The short-term impact of dolphin watching on the behavior of bottlenose dolphins (*Tursiops truncatus*) in western Australia. *Journal of Marine Animals and Their Ecology*, **2**, 3-9.

Bunbury Port Authority (2011) Bunbury Port Authority Annual Report 2011.

Department of Arts, Sports, the Environment, Tourism and Territories (1990) Nomination of Shark Bay, Western Australia by the government of Australia for inclusion in the World Heritage List. Canberra, Australia.

Department of Environment, Sport and Tourism (1995) World Heritage Information Kit, Shark Bay, Western Australia. Canberra, Australia.

Foroughirad, V. & Mann, J. (2013) Human fish provisioning has long-term impacts on the behavior and survival of bottlenose dolphins. *Biological Conservation*, **160**, 242-249.

Jensen, F.H., Bejder, L., Wahlberg, M., Aguilar Soto, N., Johnson, M. & Madsen, P.T. (2009) Vessel noise effects on delphinid communication. *Marine Ecology Progress Series*, **395**, 161-175.

Mann, J., Kemps & C. (2003) The effects of provisioning on maternal care in wild bottlenose dolphins, Shark Bay, Australia. *Marine mammals and humans: toward a sustainable balance* (eds N. Gales, M. Hindell & R. Kirkwood), pp. 304-317. Melbourne University Press. Melbourne.

Preen, A.R., Marsh, H., Lawler, I.R., Prince, R.I.T. & Shepherd, R. (1997) Distribution and abundance of dugongs, turtles, dolphins and other megafauna in Shark Bay, Ningaloo Reef and Exmouth Gulf, western Australia. *Wildlife Research*, **24**, 185-208.

Smith, H.C., Pollock, K., Waples, K., Bradley, S. & Bejder, L. (2013) Use of the robust design to estimate seasonal abundance and demographic parameters of a coastal bottlenose dolphins (*Tursiops aduncus*) population. *Plos One*, **8**, DOI:10.1371/journal.pone.0076574.

Wahlquist, C. (2011) City unprepared for population boom: Mayor. Bunbury Mail.

Walker, D.I. & Prince, R.I.T. (1987) Distribution and biogeography of seagrass species on the northwest coast of Australia. *Aquatic Botany*, **29**,19-32.

**S2 Parameters other than reproductive and survival rates**

Population sizes & carrying capacities

The Shark Bay population size was estimated as 2888 (SD 434) by repeated aerial surveys (Preen *et al*. 1997). Because both gulfs cover approximately the same area (see Fig. S1a) and the dolphin densities are similar throughout the bay (Preen *et al*. 1997), it was assumed that the two populations were approximately the same size, i.e. 1444 individuals each. Carrying capacity is inherently difficult to calculate, however it has been shown that carrying capacities based on observed population size of an unaffected population will give realistic predictions with PVA models (Brook *et al*. 1997). The carrying capacity for the east and west populations were therefore set at 2000 each, i.e. above the numbers estimated by Preen *et al*. (1997), to allow for positive population growth.

For the Bunbury population we estimated the population size based on photo-identification surveys between February 2007 and March 2010 (Smith 2012; Smith et al. 2013). Unlike Smith *et al*. (2013) and Sprogis *et al*. (2016), who reported seasonal abundances for 2007 to 2009, our estimates of population size and vital rates are for the entire three-year survey period (2007-2010). We identified a total of 259 individuals of which 243 were known to be alive throughout this three-year period. To account for any unmarked individuals that may have been missed in the census, we added an additional 10% to give an estimated population size of 267. The addition of 10% assumes that approximately 90% of the individuals in the population were accounted for. This value is derived from two other studies by our collaborators on coastal dolphins with high site fidelity and large survey effort. (1) The estimated percentage of distinctive individuals detected in three species of coastal dolphins at three different sites in Western Australia ranged between 89% and 100% (93% to 95% for bottlenose dolphins) (Brown *et al*. 2016). (2) In capture-mark recapture analyses of the Bunbury population, Sprogis *et al*. (2016) estimated similar marked percentages, ranging from 80% to 90%. The carrying capacity estimate for the Bunbury population was set at 370. This value was calculated by applying the ratio of Shark Bay carrying capacity/population size ratio (4000/2888) to the Bunbury population size.

Age classes & age class distribution

Age classes were grouped into three main classes: calf, juvenile and adult (Table S2). As a result of following them for many years, for the majority of individuals in SB we know the approximate date of birth. Consequently, for the Shark Bay dolphins, age classes could be confidently determined from dates of birth for calves and juveniles because most animals were observed within one or two years of birth. Age-class structure of the Bunbury population (2007-2010) was based primarily on known dates of birth, but also relied on body size and behavior (Smith 2012; Smith *et al*. 2013).

**Table S2** The three main age classes, calf, juvenile* and adult with corresponding ages and vortex age categories of the Shark Bay and Bunbury population.

|  |  | **Females** | | **Males** | |
| --- | --- | --- | --- | --- | --- |
| Age | Age category (vortex) | Age class* |  | Age class |  |
| 0-3 yrs | 0 | Calf |  | Calf |  |
| 3-6 yrs | 1 | (Juve-1)* |  | (Juve-1)* |  |
| 6-9 yrs | 2 | (Juve-2)* | Juveniles | (Juve-2)* | Juveniles |
| 9-12 yrs | 3 | (Sub-adult-1)* |  | (Sub-adult-1)* |  |
| 12-15 yrs | 4 | Adult |  | (Sub-adult-2)* |  |
| 15 + yrs | 5 + | Adult |  | Adult |  |

*Juvenile survival rates for the Shark Bay population were subdivided into “juve-1”, “juve-2” and “sub-adult” (in parentheses); sub-adult survival rate categories for Shark Bay males range from age 9-15 years (vortex age categories 3-4), but for females, who mature earlier, only from age 9-12 years (vortex age category 3). Corresponding ages are shown in years (yrs).

Calves were defined as individuals that had not yet been weaned by their mothers (age 0-3 years; vortex age category 0). If the date of birth was unknown, calves were determined based on size (1-1.5 m) and swimming in consistent proximity in ‘infant position’ under the peduncle and tail flukes of the mother (Smith 2012). The minimum weaning period for bottlenose dolphins is approximately 3 years (Mann *et al*. 2000).

Dolphins were considered juveniles once they had been weaned, and were no longer maintaining infant position, but were less than age 12 years for females or 15 years for males. Shark Bay juvenile survival rates were further grouped into four subclasses: juve-1 (vortex age category 1), juve-2 (vortex age category 2), sub-adult-1 (both sexes: vortex age category 3) and sub-adult-2 (only males: vortex age category 4) (Table S2).

We defined animals as adults at a time point when they typically first bear offspring (i.e. not at onset of maturity, but at a later time point)—at age 12 years (vortex age categories 4-10) for females (Mann *et al*. 2000) and age 15 years (vortex age categories 5-10) for males, based on approximate age of stable male alliance formation (Connor *et al*. 2000). If the exact age was unknown, juvenile and adult age classes were determined based on size, a commonly used indicator for bottlenose dolphin age classes (e.g. Hale *et al*. 2000; Mann *et al*. 2000)—Bunbury juveniles were classified as being approximately up to 2 m long, adults approximately 2.5 meters; SB juveniles and adults are slightly smaller. Additionally, adult females in Shark Bay were aged based on the degree of ventral speckling, because it correlates with age, and speckling onset has been associated with sexual maturity (Krzyszczyk & Mann 2012). Little or no body speckling has been observed on Bunbury bottlenose dolphins.

We set the maximum age of reproduction to age 33 years (vortex age category10) because this age is close to the oldest individual identified at Bunbury, and many females reproduce into their mid-thirties—the oldest female known to successfully reproduce in SB was 39 years old (J. M. personal communication).

Due to the fact that there is more uncertainty about the age at which males usually sire their first offspring (Krützen *et al*. 2004a; Kopps 2007), we also ran scenarios with males producing their first offspring at age 12 years (vortex age category 4). These scenarios produced the same population growth forecasts as the standard models (data not shown), so we did not make any adjustments to the standard models.

For both populations we determined the age class distribution, i.e. the proportion of calves, juveniles and adults that were photo-identified to be alive throughout a given time period (Shark Bay N = 368; Bunbury N = 243) (see Table 1 of main article). The age-class distributions, i.e. the proportions of calves, juveniles and adults, of the two populations did not differ significantly (χ^2^ contingency = 2.30, *P* = 0.317, df = 2). In line with the vortex settings, the first age category (0) was not entered in the specified age category distribution setting of the vortex model.

Dispersal rates between Shark Bay subpopulations

We calculated the number of animals dispersing between the subpopulations in the west and east gulf of Shark Bay (see Fig. S1a) as *Nm*, the effective dispersal of individuals per generation between the west and east gulf. *Nm* was derived from 10 microsatellite loci, assuming equilibrium, symmetrical dispersal and selective neutrality (Krützen *et al*. 2004a and unpublished). *Nm* was calculated from the formula: *F_st_* = 1/(1+4N*m*) where *F_st_* is the population fixation index (Wright 1951; Weir & Cockerham 1984) for the 10 microsatellites. From this calculation, dispersal between the west and east subpopulations was *Nm* = 9.79 migrants per generation. Next, generation time was calculated from our demographic dataset, in two ways: from averaging the age of the mothers at the time of birth of 170 calves in Shark Bay (JMcD, unpublished BSc Honours thesis), giving a value of 19.57 years; and from the calculation of generation time that was output by a preliminary vortex model on a single SB population without dispersal, which followed the approach described by Ricklefs (1979), giving a value of 20.7 years.  From these figures for *Nm* and generation time, the number of individuals dispersing between east and west Shark Bay per year (*Nm*/generation time), appears to be close to 0.5, so that dispersal per three-year modelled time-period was taken to be 1.5. Both the genetic calculation and the vortex modelling are for summed male and female dispersal.

The likelihood of males or females dispersing was considered to be equal, because there is little evidence of sex-biased dispersal between the two gulfs. Juveniles were considered to be the youngest animals to disperse individually, because at that age class bottlenose dolphins are weaned and cease to swim in calf position close to their mother (Mann *et al*. 2000). The age of the oldest animal to disperse was considered to be the maximum age at which bottlenose dolphins typically breed (i.e. age category10).

Sex ratios for age class distribution of initial population size

We utilized a variety of methods to estimate age-specific sex ratios. Our sex ratio estimate of the Shark Bay dolphins relied on genetic sexing by Krützen *et al*. (2003). The observed sex ratio for adults was 54:46 males to females, which is not significantly different from an equal sex ratio (50:50). Therefore, for the initial population size and age distribution of the vortex model of the Shark Bay population, we evenly distributed all age classes between the two sexes. Sex ratio estimates of the Bunbury population were determined by direct observation of genital slits (N = 10), examination of stranded carcasses (N = 7) and genetic sexing (N = 46) based on the protocol of Gilson *et al.* (1998). The sex ratio of all ages for this cohort was 45:55 males to females, which was significantly skewed toward females (χ^2^ = 21.00; *p* < 0.0001; df = 1). We thus applied this unequal sex ratio to the initial age category distribution (for all three age classes) of the Bunbury model.

Sex ratios at birth

For the vortex models of both populations, we used a 50:50 ratio as the sex ratio at birth (as specified by Lacy *et al*. 2005). This was done because, with few exceptions (Wiley & Clapham 1993), cetaceans are reported to display a 50:50 sex ratio at birth (e.g. Kasuya & Marsh 1984). There is no indication that the sex ratio of the two bottlenose dolphin populations is unequal at birth.

Mate monopolization

There is little information on the percentage of males contributing to the breeding pool in marine mammals. The information on mate monopolization, i.e. the percentage of males contributing to the gene pool for both populations, came from genetic information on the Shark Bay population. Paternity analysis allowed an estimate of the median age of first male reproduction to be made, as well as an estimate of the lower limit of the proportion of males contributing to the breeding pool (Krützen *et al*. 2004b). The proportion of sampled males that could be genetically assigned paternities (13%) was taken as the lower limit to the proportion breeding, although, when improved data on paternities accumulates, it is possible that this percentage may be much higher. In the standard model the estimate of the male contribution to the breeding pool (56.5%) was taken to be the midpoint of this large possible range (13-100%).

**References for S2**

Brook, B.W., L. Lim, R. Harden & Frankham, R. (1997) Does population viability analysis software predict the behaviour of real populations? A retrospective study on the Lord Howe Island woodhen *Tricholimnas sylvestris* (Sclater). *Biological Conservation*, **82**, 119-128.

Brown, A.M., Bejder, L., Pollock, K.H., Allen, S.J. (2016) Site-specific assessments of the abundance of three inshore dolphin species to inform conservation and management. *Frontiers in Marine Science*, doi: 10.3389/fmars.2016.00004

Connor, R.C., Wells, R., Mann, J. & Read, A. (2000) The bottlenose dolphin: social relationship in a fission-fusion society. *Ceatacean Scoieties: Field studies of whales and dolphins* (eds J. Mann, J., R.C. Connor, P. Tyack, P. & H. Whitehead), pp. 91-126. University of Chicago Press, Chicago.

Gilson, A., M. Syvanen, K. Levine & Banks, J. (1998) Deer gender determination by polymerase chain reaction: validation study and application to tissues, bloodstains, and hair forensic samples from California. *California Fish and Game*, **84**,159-169.

Kasuya, T. & Marsh, H. (1984) Life history and reproductive biology of the short-finned pilot whale, *Globicephala macrorhynchus* off the Pacific Coast of Japan. *Reproduction in whales, dolphins and porpoises: Proceedings of the conference of cetacean reproduction, estimating parameters for stock assessment and management Reports of the International Whaling Commission* (eds W.F. Perrin, R.L. Brownell & D.P. DeMaster), pp. 259-310. International Whaling Commission.

Kopps, A.M. (2007) *Paternity assessment in bottlenose dolphin (Tursiops sp.)—a species with multi-level male alliances*. Master’s thesis, University of Zurich.

Krützen, M., Sherwin, W.B., Connor, R.C., Barré, L.M. & van de Casteele, T. (2003) Contrasting relatedness patterns in bottlenose dolphins (*Tursiops* sp.) with different alliance strategies. *Proceedings of the Royal Society B*, **270**, 497-502.

Krützen, M., Sherwin, W.B., Berggren, P. & Gales, N. (2004a) Population structure in an inshore cetacean revealed by microsatellite and mtDNA analysis: bottlenose dolphins (*Tursiops* sp.) in Shark Bay, Western Australia. *Marine Mammal Science*, **20**, 28-47.

Krützen, M., Barré, L.M., Connor, R.C., Mann, J. & Sherwin, W.B. (2004b) 'O father: where art thou?' - Paternity assessment in an open fission-fusion society of wild bottlenose dolphins (*Tursiops* sp.) in Shark Bay, Western Australia. *Molecular Ecology*, **13**, 1975-1990.

Krzyszczyk, E. & Mann, J. (2012) Why become speckled? Ontogeny and function of speckling in Shark Bay bottlenose dolphins (*Tursiops* sp.). *Marine Mammal Science*, **28**, 295-307.

Lacy, R.C., Borbat, M. & Pollak, J.P. (2005) VORTEX: a stochastic simulation of the extinction process. Version 9.50. Chicago Zoological Society, Brookfield, IL., U.S.

Mann, J., Connor, R.C., Barré, L.M. & Heithaus, M.R. (2000) Female reproductive success in bottlenose dolphins (*Tursiops* sp.): life history, habitat, provisioning, and group-size effects. *Behavioral Ecology*, **11**, 210-219.

Preen, A.R., Marsh, H., Lawler, I.R., Prince, R.I.T. & Shepherd, R. (1997) Distribution and abundance of dugongs, turtles, dolphins and other megafauna in Shark Bay, Ningaloo Reef and Exmouth Gulf, western Australia. *Wildlife Research*, **24**, 185-208.

Ricklefs, R.E. (1979) *Ecology*. 2^nd^ edition, Chiron Press, New York.

Smith, H.C. (2012) *Population dynamics and habitat use of bottlenose dolphins (Tursiops aduncus) in Bunbury, Western Australia*, PhD thesis, Murdoch University.

Smith, H.C., Pollock, K., Waples, K., Bradley, S. & Bejder, L. (2013) Use of the robust design to estimate seasonal abundance and demographic parameters of a coastal bottlenose dolphins (*Tursiops aduncus*) population. *Plos One*, **8**, DOI:10.1371/journal.pone.0076574.

Sprogis, K.R., Pollock, K.H., Raudino, H.C., Allen, S.J., Kopps, A.M., Manlik, O., Tyne, J.A.

& Bejder, L. (2016a). Sex-specific patterns in abundance, temporary emigration and survival of Indo-Pacific bottlenose dolphins (*Tursiops aduncus*) in coastal and estuarine waters. *Frontiers in Marine Science,* **3**, doi: 10.3389/fmars.2016.00012.

Weir, B.S. & Cockerham C.C. (1984) Estimating F-statistics for the analysis of population structure. *Evolution*, **38**, 1358-1370.

Wiley, D.N &, Clapham, P.J. (1993) Does maternal condition affect the sex-ratio of offspring in humpback whales? *Animal Behaviour*, **46**, 321-324.

Wright, S. (1951) The genetic structure of populations. *Annals of Eugenics*, **15**, 323-354.

**S3** **Reproductive and survival rates**

We assessed and compared the vital rates, i.e. reproductive and age-specific survival rates, of the two dolphin populations. Vital rates for the Shark Bay (SB) populations were determined for four consecutive three-year time periods between 1988 and 1999. Bunbury vital rates were determined for one three-year time period between 2007 and 2010 (see main article).

**Reproductive rates**

In line with the three-year setting for the vortex models, reproductive rates are here defined as the percentage of females breeding per three-year time period. We used snapshot analyses to estimate reproductive rates per three-year interval. The snapshot analysis accounts for all individuals in the population at a given time period and assumes approximate constancy of environmental effects on demography. The reproductive rate estimate for the Bunbury population was derived from 81 adult females (2007-2010). Reproductive rates for the SB population were based on a subset of the population consisting of 43 (1988-1990), 64 (1991-1993), 70 (1994-1996) and 78 (1997-1999) adult females.

The percentage of females breeding per three-year period in SB ranged from 48.44% (1991-1993) to 72.09% (1988-1990) with a mean of 58.35% across all four three-year intervals (Fig. S3a). The three-year reproductive rate of the Bunbury population was 40.74% (2007-2010), which is lower than the lowest observed three-year reproductive rate of the SB population (1991-1993) (Fig. S3a). The fact that the four three-year SB reproductive rates (1988-1999) were significantly different from one another (χ^2^ contingency = 8.44, *P* = 0.0377, df = 3) did not allow us to directly compare the three-year Bunbury rate (2007-2010) to the mean rate of all three-year SB periods (1988-1999). Therefore, we used *q*-tests, post-hoc analyses analogous to Dunnett’s tests (Zar 1996, p. 562) to compare the Bunbury reproductive rate with each of the four three-year SB rates. Bunbury reproductive output was significantly lower than that of SB 1988-1990 (SE = 5.38, *q* = 3.36, *q*_0.05 (2), ∞, 5_ = 2.44) and SB 1994-1996 (SE = 4.66, *q* = 2.71, *q*_0.05 (2), ∞, 5_ = 2.44). Given that for Bunbury we had only one sampling window, the relatively low reproductive rate for this one three-year time period might have been an outlier. Therefore, the accuracy of the Bunbury forecast would benefit from taking additional time periods into account. We therefore encourage continuous monitoring of the Bunbury vital rates.

Age-specific survival rates are the percentages of calves, juveniles and adults surviving per three-year time period. Individuals that had been confidently identified and been surveyed annually for many years were assumed to have died once they had not been sighted for at least three years. Calves under three years of age were presumed dead if there were two or more sightings of the mother without the calf. Survival rates were calculated from 274 individuals in the Bunbury population (2007-2009) and an average of 346.83 individuals across four three-year time periods in Shark Bay (1988-1999). Juvenile survival rates of the Shark Bay population were further subdivided into juve-1, juve-2 and sub-adult survival rates (see Table 1 of main article; Table S2). Three-year vital rates of both populations are shown in Fig. S3.

For the standard models of both populations we set survival rates to be equal in both sexes. Additionally, we investigated the sensitivity of the SB-model to the possibility that juvenile survival rates are higher in females (Stanton & Mann 2012). If juvenile survival rates were skewed toward females, the SB population was forecast to be more stable (*r* = 0.009) compared to the SB standard model forecast (*r* = 0.005) (see main text; Table S.5). Sexing of carcasses did not indicate that survival in the Bunbury population is sex-biased (15 mortalities; χ^2^ = 0.067; *P* = 0.80; df = 1). The vortex program requires mortality rates, so we transformed survival rates to mortality rates (mortality = 100 – survival). However, our results hereafter are presented as survival rates.

**Survival Rates**

In contrast to reproductive rates, there was little difference in survival rates between the two populations (Table S3; Fig. S3). SB juvenile survival rates (97.2%) were higher than those of the Bunbury population (90.9%) (χ^2^ contingency = 6.95, *P* = 0.008, df = 1), whereas SB adult survival was lower (SB: 90.3%, Bunbury: 95.9%; χ^2^ contingency = 4.57, *P* = 0.03, df = 1) (Table S3; Fig. S3c-d). There was neither a significant difference in calf survival rates (SB: 73.5%; Bunbury: 71.7%; χ^2^ contingency = 0.11, *P* = 0.74, df = 1), nor in non-calf (i.e. juvenile & adult combined) survival rates (SB: 93.6%; Bunbury: 94.4%; χ^2^ contingency = 0.003, *P* = 0.96, df = 1). This similarity in survival rates between the two populations is consistent with data derived using CMR methodology that show identical non-calf survival rates of 95.0% for both populations (Nicholson *et al*. 2012; Smith *et al*. 2013) (Table S3). See also Appendix S4 for applicability of capture-mark-recapture (CMC) methodology.

**Table S3** Survival rate comparison between Shark Bay and Bunbury. Survival rates for this study are listed as the percentage of survivors per three-year period with the mean values given for Shark Bay (for four three-year time periods). We also list non-calf survival rates based on capture-mark-recapture methods (Nicholson *et al*. 2012; Smith *et al*. 2013). Contingency Chi-square values (χ^2^) for the comparison of Shark Bay and Bunbury survival rates, as well as associated degrees of freedom (df) and *p* values.

| **Survival rates** | **Shark Bay** | **Bunbury** | **χ^2^** | **df** | ***P*** |
| --- | --- | --- | --- | --- | --- |
| Calf survival | 73.5% | 71.7% | 0.107 | 1 | 0.744 |
| Juvenile survival | 97.2% | 90.9% | 6.951 | 1 | 0.008 |
| Adult survival | 90.3% | 95.9% | 4.668 | 1 | 0.033 |
| Non-calf survival | 93.6% | 94.4% | 0.003 | 1 | 0.956 |
| Non-calf survival: | 95.0%* | 95.0%† | NA | NA | NA |
| CMR-based |  |  |  |  |  |

*Nicholson *et al*. 2012; †Smith *et al*. 2013

**Variation of reproductive and survival rates**

To determine vortex input parameters and to assess the relative degree of temporal variation of vital rates, we determined standard deviations due to environmental variances (SD_EV_). The total observed variance in each vital rate is the sum of stochastic variance and variance due to environmental fluctuations. Hence, to obtain SD_EV_s, we subtracted the expected binomial sampling variance from the total observed temporal fluctuations following Miller and Lacy (2005). We also calculated the associated coefficients of variation (CV), i.e. the ratio of the standard deviation to the mean rates (see Table 1 of main article). The dimensionless coefficients of variation allowed comparisons of variation in reproductive and survival rates. For Shark Bay we compared variation in vital rates on the basis of three-year CVs. For Bunbury, lacking long-term data, we determined Bunbury annual vital rates and associated SDs and CVs to assess temporal variation in vital rates. Although the annual rates (and associated SDs & CVs) were not used in our three-year vortex model, they allowed us to compare the relative variability of each vital rate for Bunbury. For the three-year vortex model we derived Bunbury three-year CVs from Shark Bay data (see *Materials and methods* in main article).

The large difference in observed temporal variation of Shark Bay reproductive versus survival rates is apparent in Fig. S3. Reproduction displayed the largest observed, temporal variation (Table 1 in main article; Fig. S3). For both populations, standard deviations and associated coefficients of variation were highest for reproductive rates, followed by calf, juvenile and adult survival rates (see Table 1 in main article). For SB, the three-year CV_EV_ for reproduction (CV_EV_ = 0.161) was substantially greater than the respective values for survival rates (CV_EV_ = 0.012-0.046) (Table 1 in main article). For Bunbury we compared temporal variation of reproduction and survival using Bunbury annual rates, because the Bunbury three-year CV_EV_s were derived from SB data (see methods, main article). Annual variation in Bunbury reproductive rates (CV = 0.636) was higher than annual variation in survival rates (CV = 0.010-0.076) (Table 1 in main article).


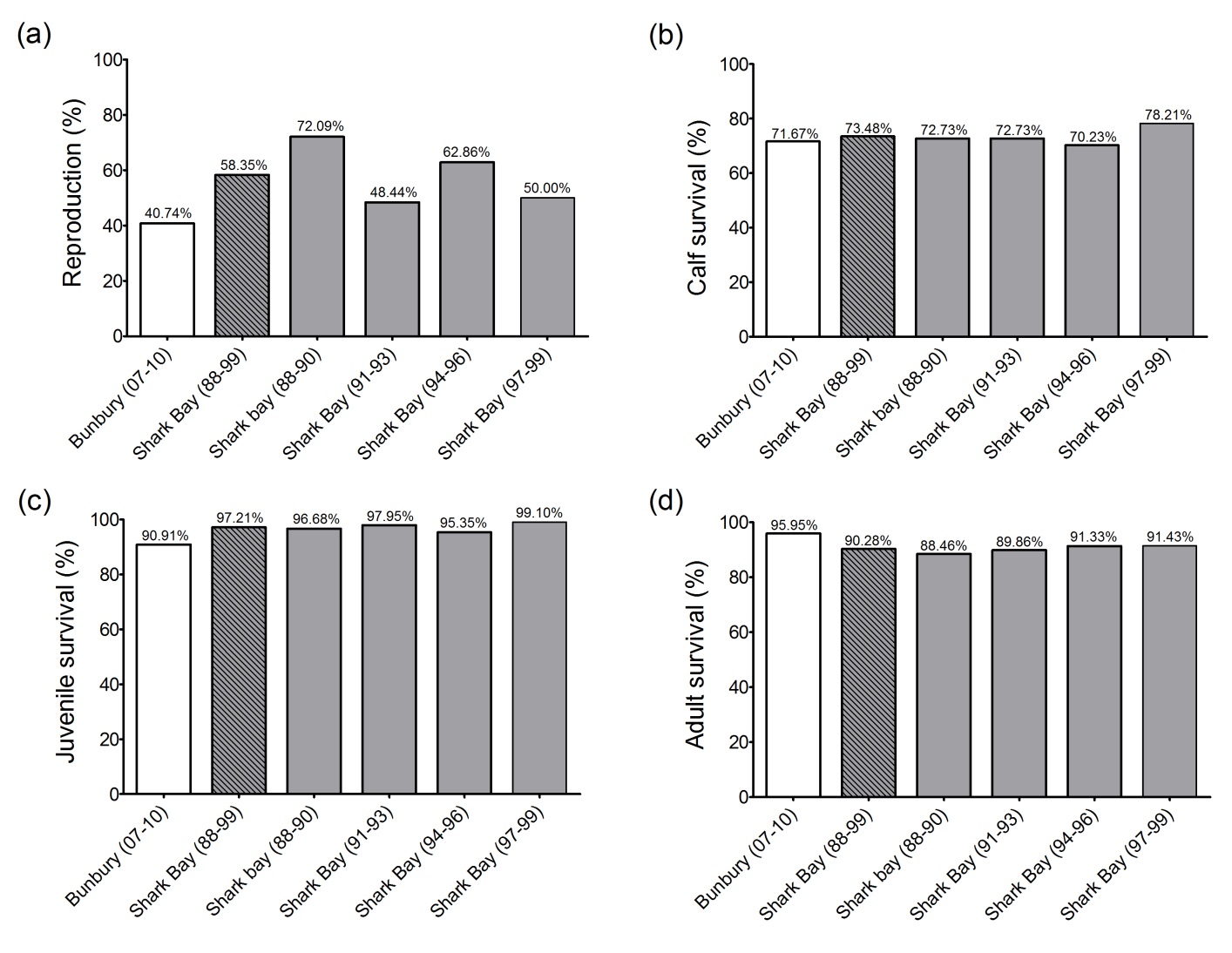


**Fig. S3.** Vital rates of Shark Bay (shaded bars) and Bunbury (white bars). For each panel, the shaded, striped bars represent the Shark Bay means for the four 3-year time periods. (a) Reproductive rates, i.e. the percentage of adult females breeding per three-year time period are shown in this panel. (b-d) Three-year survival rates for calves (b), juveniles (c) and adults (d).

**References for S3**

Miller, P.S. & Lacy, R.C. (2005) VORTEX. A stochastic simulation of the extinction process. Conservation Breeding Specialist Group (IUCN/SSC), Apple Valley, Minnesota.

Stanton, M.A. & Mann, J. (2012) Early social networks predict survival in wild bottlenose dolphins. *Plos One*, **7**, DOI:10.1371/journal.pone.0047508.

Zar, J.H. (1996) *Biostatistical Analysis*. Prentice Hall, New Jersey.

**S4 Applicability of capture-mark-recapture methodology**

Due to the high survey efforts we were able to confidently estimate age-specific demographic parameters without having to rely on capture-mark-recapture (CMR) methods. CMR methodology would also allow us to estimate survival rates for juveniles and adults—as done by Nicholson *et al*. (Shark Bay) (2012) and Smith *et al*. (Bunbury) (2013). However, using CMR-derived survival rates was less suitable for our comparative analysis because it was not possible to use CMR to reliably estimate calf survival rates of both populations. The reason for this is that calves are—at least for the Bunbury population—not yet sufficiently marked, i.e. they lack distinctive dorsal fin markings (H. Smith & K. Nicholson personal communication). Given that PVA modelling requires survival rates for all age classes, calf survival cannot be neglected, especially because calf survival rates are typically much lower than those of non-calves. Therefore, in order to compare population dynamics of the two populations—which requires using the same reliable methodology for both populations—we did not use CMR-derived survival rates for the standard models.

Nevertheless, we compared our survey-based survival rates with published survival rates on the basis of CMR methods for Bunbury (Smith *et al*. 2013), the Western Gulf of Shark Bay (Nicholson *et al*. 2012) and the Eastern Gulf of Shark Bay (unpublished data; Krzyszczyk 2013; personal communication E. Krzyszczyk). We also compared the effect on model forecasts, when using our survey-based survival rates (i.e. standard model) versus scenarios that use CMR-derived survival estimates.

Bunbury (Smith *et al*. 2013) and Shark Bay (Nicholson *et al*. 2012) CMR-based annual survival estimates for non-calves (i.e. adults and juveniles) were both reported to be 95% (SE 0.02) (As mentioned above, the authors did not estimate survival rates for calves.) Using the three-year survival estimates from our surveys (which we applied to the standard models), we also did not find a difference between non-calf survival rates for the Shark Bay (93.6%) and Bunbury population (94.4%) (χ^2^ contingency = 0.003, *P* = 0.9561, df = 1). Our non-calf survey-based annual mean survival rate estimates for Shark Bay (97.8%) and Bunbury (98.1%) are higher than the CMR-derived annual rates reported by Nicholson *et al*. 2012 (West Shark Bay: 95.0%) and Smith *et al*. (2013) (Bunbury: 95.0%). However, they are within the 95% confidence limits (West Shark Bay & Bunbury: 91.1% to 98.9%) of the published CMR-based estimates.

In order to compare the effect of various survival rate estimates on the forecast for population dynamics we modelled two scenarios: (1) CMR-adult survival and (2) CMR-non-calf survival. For the CMR-adult survival we used adult survival rate estimates based on CMR methods (Shark Bay: Nicholson *et al*. 2012; Bunbury: Smith *et al*. 2013), but relied on our survey-based juvenile and calf survival rate estimates. For the CMR-non-calf survival scenario we applied the non-calf (i.e. juvenile & adult combined) survival rates reported by Nicholson *et al*. (2012) and Smith *et al*. (2013).

In comparison to the standard models, using survival rates estimated with CMR methods resulted in different forecasts, but did not change the overall findings, which showed a large difference in the viability of the two populations—with a relatively stable Shark Bay population and an apparently declining Bunbury population that is at high risk of extinction (Fig. S4). Applying CMR-based adult survival rates to the Shark Bay model forecast a higher growth rate (*r* = 0.011)—relative to the standard model (*r* = 0.005)—but using CMR-non-calf survival rates resulted in slightly negative growth (*r* = – 0.0059). In each scenario the probability of extinction for the 300-year forecast of the Shark Bay population was 0%. In comparison to the Bunbury standard model, application of CMR-based survival rates forecast a steeper decline of the Bunbury population (CMR-adult survival: *r* = – 0.071; CMR-non-calf survival: *r* = – 0.069) with a probability of extinction of 99% (compared to 96% for the standard model).

Regardless of which survival rates we used—CMR-based or the survey-based survival rates of the standard model—the scenarios all showed a big difference in viability of the two populations (Fig. S4). This was also the case when using non-calf survival rate estimates based on a 25-year CMR analysis of the Shark Bay population (unpublished data; Krzyszczyk 2013; personal communication E. Krzyszczyk).


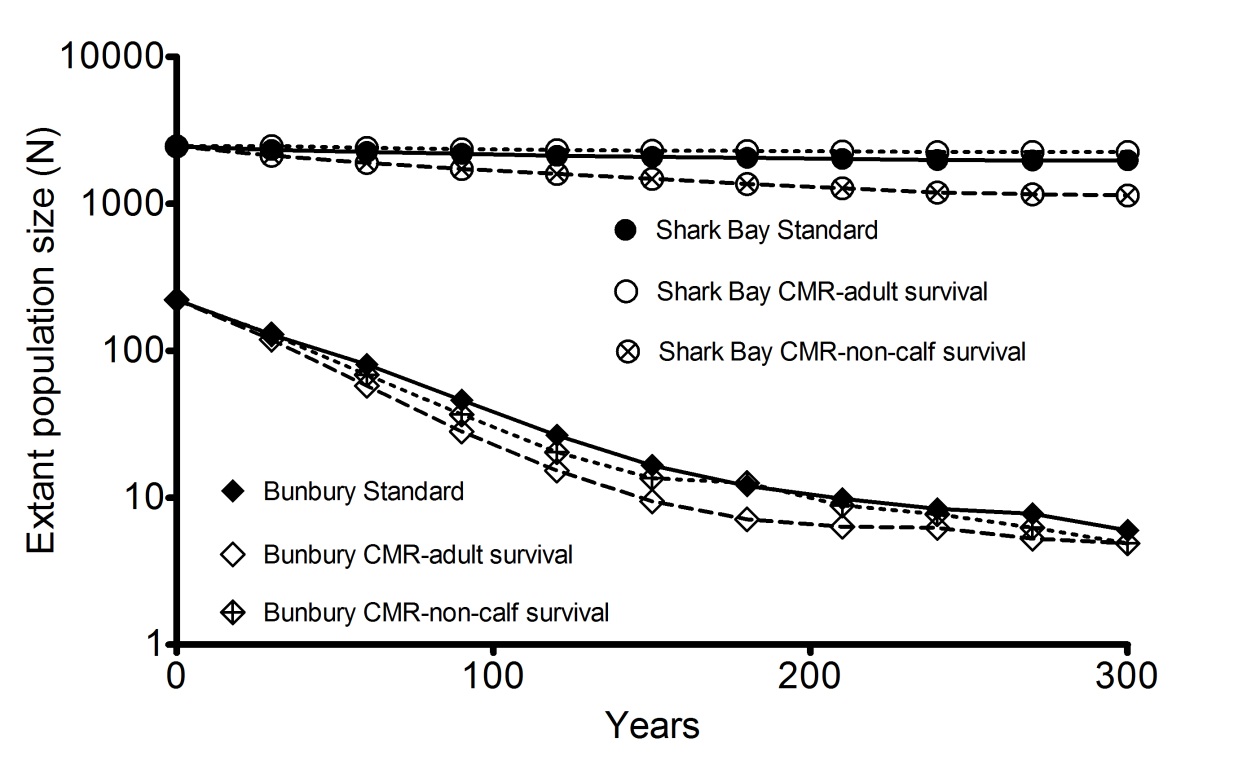
 The fact that both the survey-based method (used in the standard models) and the CMR-based method (Shark Bay: Nicholson *et al*. 2012; Bunbury: Smith *et al*. 2013) show no significantly different non-calf survival rates between the two populations, supports our finding that this difference in viability between the two populations is not due to survival. Thus, any conceivable bias in our methodology to estimate survival rates (e.g. due to non-detection of animals) would not affect our findings in respect to the relative importance of survival versus reproduction.

**Fig. S4.** Comparison of standard model forecasts with forecasts from scenarios using adult and non-calf (i.e. juvenile & adult combined) survival rates estimated from capture-mark-recapture methodology (based on CMR estimates from Nicholson *et al*. 2012, and Smith *et al*. 2013).

**References for S4**

Krzyszczyk, E. (2013) *Age determination, life history and juvenile behavior in bottlenose dolphins (Tursiops sp.) in Shark Bay, Australia*, PhD thesis, Georgetown University.

Nicholson, K., Bejder, L., Allen, S.J., Krützen, M. & Pollock, K.H. (2012) Abundance, survival and temporary emigration of bottlenose dolphins (*Tursiops* sp.) off Useless Loop in the western gulf of Shark Bay, Western Australia. *Marine and Freshwater Research*, **63**, 1059-1068.

Smith, H.C., Pollock, K., Waples, K., Bradley, S. & Bejder, L. (2013) Use of the robust design to estimate seasonal abundance and demographic parameters of a coastal bottlenose dolphins (*Tursiops aduncus*) population. *Plos One*, **8**, DOI:10.1371/journal.pone.0076574.

**S5 Results of Standard Models**

**Table S5** Forecasts for stochastic growth rate (*r*), 100-year and 300-year population size (*N_100_*, *N_300_*) and retention of genetic diversity (*Gdiv._100_*, *Gdiv._300_*) of the Shark Bay and Bunbury standard models.

|  |  | **Shark Bay** | **Bunbury** |
| --- | --- | --- | --- |
|  | Growth rate (*r*) | 0.005 (SE 0.0001) | – 0.061 (SE 0.0005) |
|  | Mean time to extinction | NA | 203 years (SE 1.32) |
|  | Extant population size (*N_100_*) | 2162 (SE 11.99) | 38 (SE 0.57) |
| **100-year forecast** | Retention of genetic diversity (*Gdiv._100_*) | 0.998 (SE 0.000) | 0.941 (SE 0.001) |
|  | Probability of extinction (*PE_10_*_0_) (%) | 0.000 | 0.000 |
|  | Extant population size (*N_300_*) | 1980 (SE 13.91) | 6 (SE 0.61) |
| **300-year forecast** | Retention of genetic diversity (*Gdiv._300_*) | 0.994 (SE 0.000) | 0.569 (SE 0.023) |
|  | Probability of extinction (*PE_300_*) (%) | 0.000 | 96 (SE 0.65) |

**S6 Elasticity analysis**

Population growth rate is determined by vital rates such as survival and reproductive rates. The relative effect of each vital rate on growth rate is commonly assessed by elasticity analysis, which is a type of fixed-proportion analysis. Elasticity is a measure of relative changes in population growth rate as a response to small proportional perturbations in vital rates (Caswell *et al*. 1984; de Kroon *et al*. 1986; de Kroon *et al*. 2000). Elasticities, i.e. proportional sensitivities, are thus regarded as the “relative contribution of a demographic parameter to population growth rate” (de Kroon *et al*. 2000). Elasticity calculations are usually based on matrix projections models and quantify the effect of changes in matrix elements (i.e. reproductive and survival rates) on the log of deterministic population growth rate, but have also been applied to a variety of stochastic scenarios (Åberg 2009).

In addition to our statistical approach, in which we applied Kruskal-Wallis (described in main article), we used elasticities to assess the effect of proportional changes in reproductive and survival rates on vortex growth rate projections. Our calculations were based on the equation by de Kroon *et al*. (1986; 2000), which describes elasticity, *e_i_*, as a measure of proportional change in discrete growth rate, λ (λ = *e*^r^, where r represents the continuous growth rate) resulting from an infinitesimal proportional change in each vital rate input, *a_i_*:

$$\text{e}\text{i}= \frac{\partial(\log\lambda)}{\partial(\log\text{a}\text{i})}$$

We calculated elasticities by changing vortex input for reproduction, calf, juvenile and adult survival from medium (standard value) to low (– 1%) and the resulting changes in discrete growth rate (λ) projections. We ranked the relative contribution of each vital rate according to their elasticity values.

The elasticity values of each population show that proportional reductions (– 1%) in adult and juvenile survival resulted in the relative greatest changes in growth rate projections (Table S6). For the Shark Bay population, juvenile survival (*e_juv._* = 0.4376) had the relatively greatest effect on population growth rate, closely followed by adult survival (*e_adult_* = 0.3884), then reproduction (*e_repro_*_._ = 0.1145) and calf survival (*e_calf_* = 0.1442). For the Bunbury population, the relative contribution to population growth was largest for adult survival (*e_adult_* = 0.4124), followed by juvenile (*e_juv._* = 0.3963) and calf survival (*e_calf_* = 0.1279); proportional changes in reproduction had the least effect on growth rate projections (*e_repro_*_._ = 0.1175). These rankings on the basis of elasticities agree with the rankings based on Kruskal-Wallis *H*-values for the fixed-proportion analysis (see Table 2 in main article). This is to be expected, given that elasticity analysis is based on proportional perturbations of vital rate inputs.

**Table S6** Elasticity values and corresponding ranking of vital rates (reproductive rates, calf, juvenile and adult survival rates)

|  | **Shark Bay** | |  | **Bunbury** | |
| --- | --- | --- | --- | --- | --- |
| Vital rates | Elasticities | Rank |  | Elasticities | Rank |
| Reproduction | 0.1445 | 3 |  | 0.1175 | 4 |
| Calf survival | 0.1442 | 4 |  | 0.1279 | 3 |
| Juvenile survival | 0.4376 | 1 |  | 0.3963 | 2 |
| Adult survival | 0.3884 | 2 |  | 0.4124 | 1 |

**References for S6**

Åberg, P., Svensson, C.J., Caswell, H. & Pavia, H. (2009) Environment-specific elasticity and sensitivity analysis of the stochastic growth rate. *Ecological Modelling*, **220**, 605-610.

Caswell, H., Naiman, R.J. & Morin, R. (1984) Evaluating the consequences of reproduction in complex salmonid life-cycles. *Aquaculture*, **43**, 123-134.

de Kroon, H., Plaisier, A., van Groenendael, J. & Caswell, H. (1986) Elasticity: the relative contribution of demographic parameters to population growth rate. *Ecology*, **67**, 1427-1431.

de Kroon, H., van Groeenendael, J. & Ehrlén, J. (2000) Elasticities: a review of methods and model limitations. *Ecology*, **81**, 607-618.

**S7 Fixed-proportion and observed-variation analysis—effect on population size forecasts (*N_100_*).**

In addition to assessing the effect of parameter perturbations on growth rate projections we also assessed the effect on 100-year population size forecasts (*N_100_*) (see Methods section and Table 2 in main text). The relative effect of parameter perturbations on 100-year population size forecasts is depicted in Fig. S7.

**
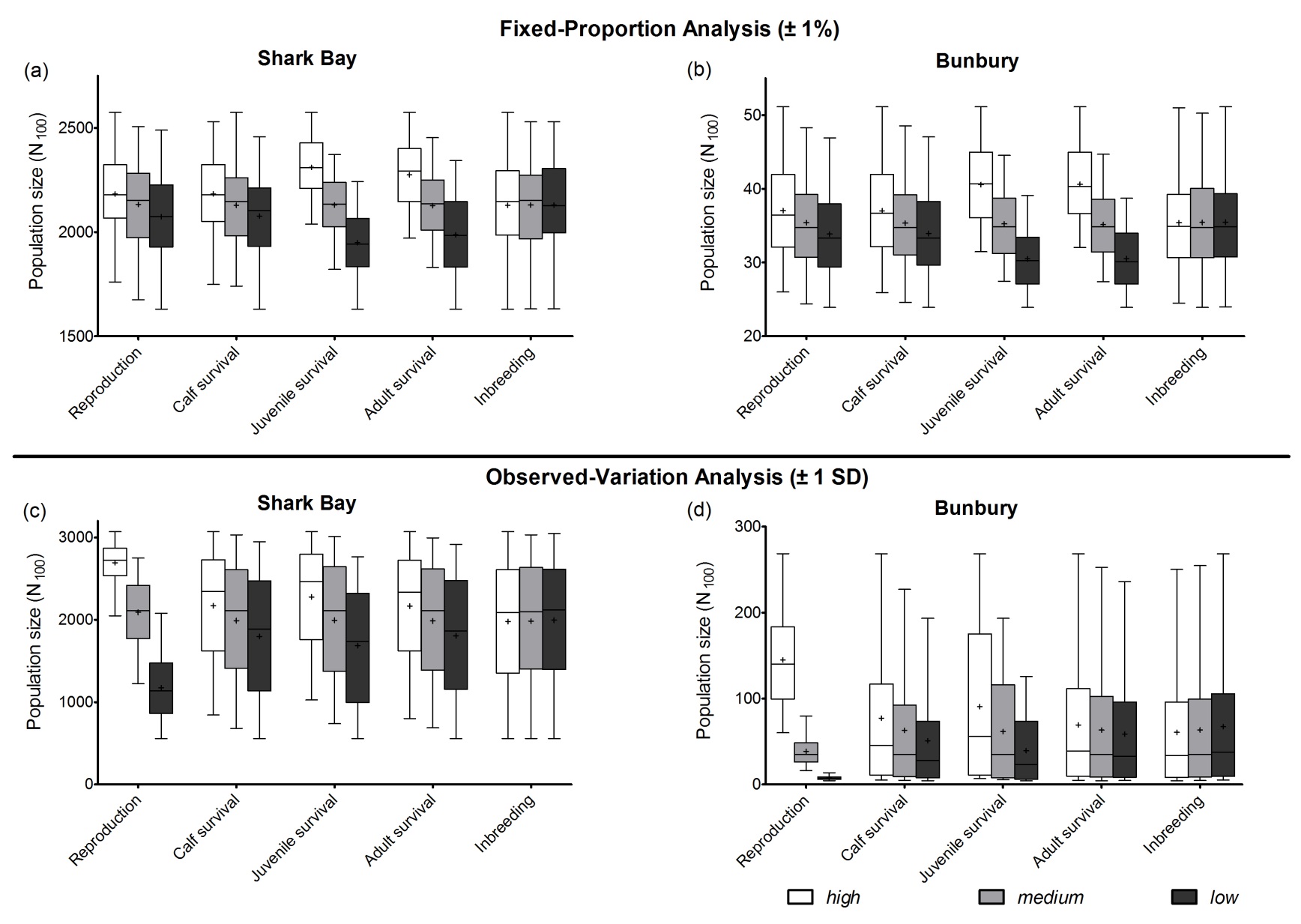
**

**Fig. S7.** Sensitivity analyses. Panels a, b: relative effect of proportional perturbations (standard value ± 1%; 3.14 lethal equivalents ± 1%) of variables on population size (N_100_). Panels c, d: relative effect of observed, temporal variation of reproductive rates and survival rates (standard value, ± 1 SD_EV_), as well as perturbations of inbreeding levels (0, 3.14, 6.28 lethal equivalents) on 100-year population size (N_100_) forecasts. Each box plot shows median, upper and lower quartile population size forecasts of 81 simulations across all (3^4^) combinations. The white, grey and dark-shaded boxes show the output from scenarios run with high, medium and low input values, respectively. Whiskers display minimum and maximum output values.

**S8 Population size forecasts with associated standard error for Shark Bay standard, Bunbury standard and forecasts based on scenarios with substituted vital rates**

**Table S8** Population size forecasts for each time period plotted in Fig. 1 of main text for a) Shark Bay standard, Shark Bay model with Bunbury survival rates, and Shark Bay model with Bunbury reproductive rates; b) Bunbury standard, Bunbury model with Shark Bay survival rates, and Bunbury model with Shark Bay reproductive rates. Associated standard errors for each population size forecast are shown in brackets.

| a) Shark Bay model with Bunbury input | | |  |
| --- | --- | --- | --- |
| **Years** | **Standard** | **Bunbury survival** | **Bunbury reproduction** |
| 30 | 2329.24 (7.82) | 2169.85 (7.15) | 1507.00 (7.42) |
| 60 | 2253.73 (9.28) | 1975.62 (8.83) | 936.76 (6.92) |
| 90 | 2188.88 (9.94) | 1836.71 (9.70) | 590.00 (5.47) |
| 120 | 2126.15 (10.37) | 1718.21 (10.40) | 367.98 (4.05) |
| 150 | 2088.03 (10.93) | 1615.58 (10.64) | 228.41 (3.06) |
| 180 | 2057.68 (11.26) | 1522.46 (10.59) | 142.11 (2.14) |
| 210 | 2019.97 (11.46) | 1452.89 (10.93) | 88.71 (1.55) |
| 240 | 1984.55 (11.56) | 1389.52 (11.20) | 56.17 (1.08) |
| 270 | 1976.12 (12.12) | 1334.45 (11.37) | 37.01 (0.81) |
| 300 | 1980.00 (11.68) | 1280.93 (11.35) | 24.88 (0.61) |
|  |  |  |  |
| b) Bunbury model with Shark Bay input | | |  |
| **Years** | **Standard** | **Shark Bay survival** | **Shark Bay reproduction** |
| 30 | 129.00 (0.86) | 147.79 (0.88) | 226.57 (0.95) |
| 60 | 80.74 (0.78) | 89.96 (0.92) | 213.59 (1.42) |
| 90 | 46.21 (0.64) | 54.68 (0.78) | 203.65 (1.74) |
| 120 | 26.56 (0.49) | 33.45 (0.61) | 194.65 (2.03) |
| 150 | 16.59 (0.39) | 21.8 (0.47) | 184.23 (2.22) |
| 180 | 12.02 (0.34) | 14.62 (0.39) | 174.54 (2.37) |
| 210 | 9.85 (0.35) | 11.76 (0.37) | 165.54 (2.54) |
| 240 | 8.41 (0.38) | 9.72 (0.39) | 157.53 (2.68) |
| 270 | 7.79 (0.50) | 8.71 (0.51) | 149.99 (2.66) |
| 300 | 6.04 (0.61) | 8.05 (0.56) | 143.01 (2.76) |

**S9 Effect of varying reproductive rates versus varying all age-specific survival rates (± 1 SD_EV_) on population trajectories**

We also conducted an observed-variation analysis in which we compared the effect of changing reproductive rates versus all age-specific survival rates simultaneously by one standard deviation (± 1 SD_EV_).

**Methods**

In this analysis, instead of varying calf, juvenile and adult survival rates separately, we varied all age-specific survival rates simultaneously. Unlike the method described for the observed-variation analysis in the main article, in which we tested all possible combinations of five parameter values, in this analysis we only ran four different scenarios: (1) high reproduction (standard value + 1 SD_EV_), (2) low reproduction (standard value – 1 SD_EV_), (3) high survival (standard values of all age-specific survival rates + 1 SD_EV_) and (4) low survival (standard values of all age-specific survival rates – 1 SD_EV_). Like the standard models and all scenarios in the sensitivity analyses described in the main article, all four scenarios were repeated for 1000 iterations.

We assessed the effect of these parameter variations on stochastic growth rate (*r*), 100- and 300-year population size forecasts (*N_100_* and *N_300_*). For that purpose we compared the growth rate and population size output means of the 1000 iterations for each of the four scenarios. Mann-Whitney, a non-parametric test, was used here because the distributions departed from normality.

**Results**

Varying reproductive rates based on natural variation (± 1 SD_EV_) had a greater effect on population dynamics than varying all age-specific survival rates simultaneously (Fig. S9; Table S9). For instance, the Shark Bay *N_300_*-forecast was significantly lower when reducing reproductive rates compared to lowering all age-specific survival rates by one SD_EV_ (Mann-Whitney *U*: 337720, *p* < 0.0001) (Fig. S9a; Table S9). Conversely, increasing reproduction of the Bunbury population by one SD_EV_ resulted in a significantly greater *N_300_*-forecast than simultaneously increasing all age-specific survival rates by one SD_EV_ (Mann-Whitney *U*: 85630, *p* < 0.0001) (Fig. S9b; Table S9).

**Table S9** Effect of changing reproductive rates versus changing all age-specific survival rates simultaneously by one standard deviation (± 1 SD_EV_) on stochastic growth rate (*r*)_,_ 100-year and 300-year population size forecasts (*N_100_* and *N_300_*). Standard errors are shown in parentheses.

|  |  | *r* | *N_100_* | *N_300_* |
| --- | --- | --- | --- | --- |
|  | Standard | 0.005 (0.0001) | 2162 (12.0) | 1980 (13.9) |
|  | Low survival (– 1 SD_EV_) | -0.018 (0.0002) | 1257 (11.1) | 414 (7.2) |
| **Shark Bay** | Low reproduction (– 1 SD_EV_) | -0.022 (0.0001) | 1150 (8.7) | 289 (4.2) |
|  | High survival (+ 1 SD_EV_) | 0.027 (0.0001) | 2743 (13.8) | 2722 (14.2) |
|  | High reproduction (+ 1 SD_EV_) | 0.028 (0.0001) | 2748 (13.3) | 2732 (14.3) |
|  | Standard | -0.061 (0.0005) | 38 (0.6) | 6 (0.6) |
|  | Low survival (– 1 SD_EV_) | -0.084 (0.0006) | 18 (0.3) | 8 (3.0) |
| **Bunbury** | Low reproduction (– 1 SD_EV_) | -0.112 (0.0007) | 8 (0.2) | 0.000 |
|  | High survival (+ 1 SD_EV_) | -0.039 (0.0003) | 79 (1.0) | 14 (0.5) |
|  | High reproduction (+ 1 SD_EV_) | -0.017 (0.0002) | 144 (1.8) | 60 (1.5) |

**
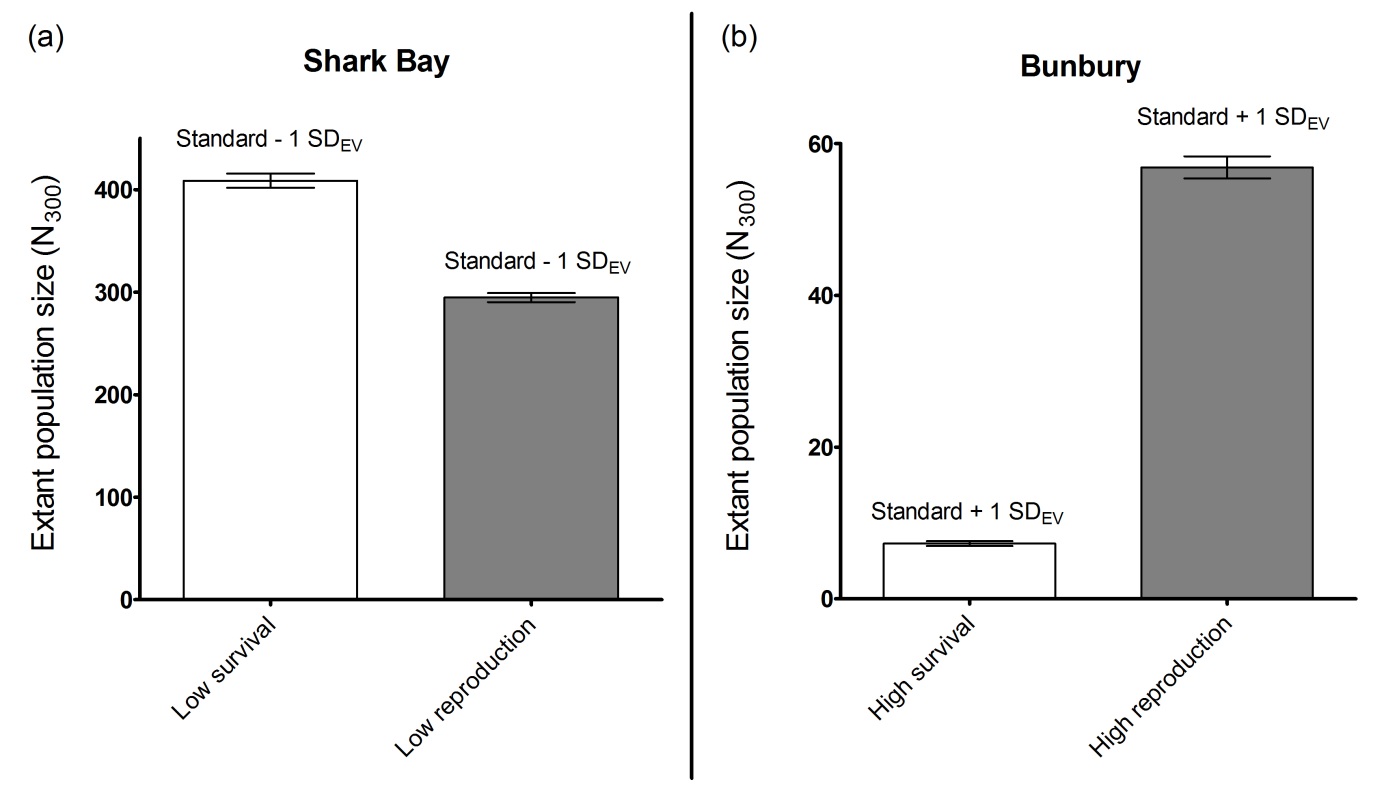
**

**Fig. S9.** (a) Effect of lowering reproductive rates versus lowering all age-specific survival rates simultaneously by one standard deviation (– 1 SD_EV_) on Shark Bay 300-year population size forecast (N_300_). (b) Effect of increasing reproductive rates versus increasing all age-specific survival rates simultaneously by one standard deviation (+1 SD_EV_) on Bunbury 300-year population size forecast (N_300_). In comparison, the 300-year population size forecasts of the standard models for Shark Bay and Bunbury were1980 (SE 13.9) and 6 (SE 0.6), respectively.
